# Unexplored Character Diversity in Onychophora (Velvet Worms): A Comparative Study of Three Peripatid Species

**DOI:** 10.1371/journal.pone.0051220

**Published:** 2012-12-17

**Authors:** Ivo de Sena Oliveira, Franziska Anni Franke, Lars Hering, Stefan Schaffer, David M. Rowell, Andreas Weck-Heimann, Julián Monge-Nájera, Bernal Morera-Brenes, Georg Mayer

**Affiliations:** 1 Animal Evolution and Development, Institute of Biology, University of Leipzig, Leipzig, Germany; 2 Division of Evolution, Ecology and Genetics, Research School of Biology, Australian National University, Canberra, Australia; 3 Senckenberg Natural History Collections, Museum of Zoology, Dresden, Germany; 4 Biología Tropical, Universidad de Costa Rica, San José, Costa Rica; 5 Laboratorio de Genética Evolutiva, Escuela de Ciencias Biológicas, Universidad Nacional, Heredia, Costa Rica; 6 Centro de Investigaciones en Estructuras Microscópicas (CIEMic), Universidad de Costa Rica, San José, Costa Rica; Sars International Centre for Marine Molecular Biology, Norway

## Abstract

Low character variation among onychophoran species has been an obstacle for taxonomic and phylogenetic studies in the past, however we have identified a number of new and informative characters using morphological, molecular, and chromosomal techniques. Our analyses involved a detailed examination of *Epiperipatus biolleyi* from Costa Rica, *Eoperipatus* sp. from Thailand, and a new onychophoran species and genus from Costa Rica, *Principapillatus hitoyensis* **gen. et sp. nov.**. Scanning electron microscopy on embryos and specimens of varying age revealed novel morphological characters and character states, including the distribution of different receptor types along the antennae, the arrangement and form of papillae on the head, body and legs, the presence and shape of interpedal structures and fields of modified scales on the ventral body surface, the arrangement of lips around the mouth, the number, position and structure of crural tubercles and anal gland openings, and the presence and shape of embryonic foot projections. Karyotypic analyses revealed differences in the number and size of chromosomes among the species studied. The results of our phylogenetic analyses using mitochondrial *COI* and *12S rRNA* gene sequences are in line with morphological and karyotype data. However, our data show a large number of unexplored, albeit informative, characters in the Peripatidae. We suggest that analysing these characters in additional species would help unravel species diversity and phylogeny in the Onychophora, and that inconsistencies among most diagnostic features used for the peripatid genera in the literature could be addressed by identifying a suite of characters common to all peripatids.

## Introduction

The putative low morphological diversity among species of Peripatidae has long been recognised as one of the main obstacles for studies of onychophoran taxonomy [1]–[3]. However, evidence suggests that this diversity has not been explored sufficiently and several structures reported in the literature have been typically neglected by taxonomists. These include, for example, modified dermal papillae [4], [5], bean-shaped leg papillae [6]–[8] and embryonic foot projections, which differ among the species studied [9]–[12]. Furthermore, chromosomal variation has been reported from many species of Peripatopsidae [13]–[15], whereas the chromosome number has been reported for only one species of Peripatidae [16]. Hence, a comparative study of these and additional features might increase the number of useful characters for studies of onychophoran taxonomy and phylogeny, and provide some consistency in the choice of diagnostic characters for new taxa.

We therefore applied morphological, molecular and karyotyping methods to clarify character variation in two closely related species of the neotropical Peripatidae from Costa Rica, and a distantly related species of the Asian Peripatidae from Thailand. To search for novel structures, we screened embryos and specimens of a range of ages using scanning electron microscopy. In addition, following previous suggestions [17]–[19], we sequenced the mitochondrial *cytochrome c oxidase subunit I* (*COI*) and *small ribosomal subunit RNA* gene sequences (*12S rRNA*) and performed karyotype analyses to clarify the genetic and chromosomal variation in the three species studied. Based on the new data, we describe a new genus and species from Costa Rica, which we have bred in the laboratory for seven years, and discuss inconsistencies regarding the diagnostic features used for the genera of Peripatidae.

## Materials and Methods

### Animals

**Figure 1:**
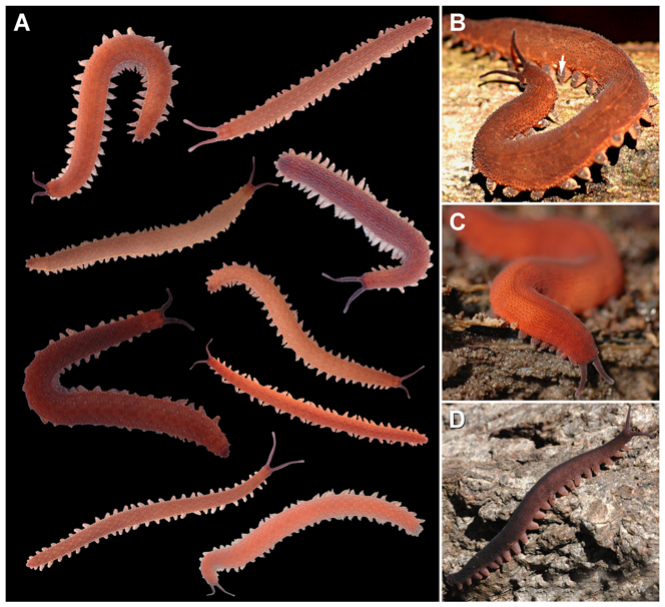
Habitus and body colour in the three species studied. Images not to scale (the size of photographed specimens ranges from 35 to 85 mm). (A) Panel demonstrating variation of colour pattern in specimens of *Principapillatus hitoyensis* **gen. et sp. nov.** (B) Specimen of *Principapillatus hitoyensis* **gen. et sp. nov.** Note dark colour of integument and a single bright papilla on dorsal leg surface (arrow). (C) Specimen of *Epiperipatus biolleyi.* (D) Specimen of *Eoperipatus* sp.

**Figure 2:**
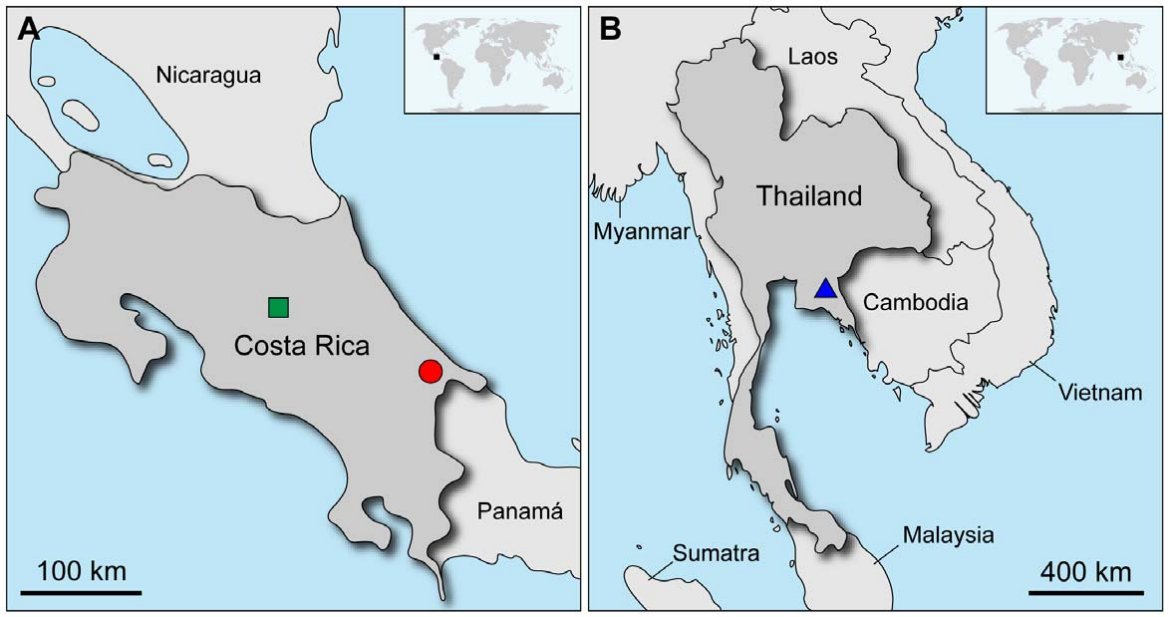
Geographic distribution of the three species studied. (A) Map of Costa Rica illustrating the type locality of *Principapillatus hitoyensis* **gen. et sp. nov.** in the Reserva Biológica Hitoy Cerere (red circle) and the collecting site of *Epiperipatus biolleyi* in Cascajal de Coronado (green square). (B) Map of Thailand indicating the location of the Chanthaburi Mountain Range (blue triangle) from which specimens of *Eoperipatus* sp. were obtained.

Three species of Peripatidae were studied (Figure 1A–D). Specimens of *Principapillatus hitoyensis* **gen. et sp. nov.** were obtained in October 2005 in the Reserva Biológica Hitoy Cerere, Province of Limón, region of Talamanca, Costa Rica (Figure 2A; 09°40′21.56″N, 83°02′36.97″W, 300 m). Specimens of *Epiperipatus biolleyi* (Bouvier, 1902) were obtained as described previously [20] in Los Juncos, Cascajal de Coronado, Province of San José, Costa Rica (Figure 2A; 10°01′27.62″N, 83°56′30.26″W, 1760 m). Specimens of *Eoperipatus* sp. were obtained in September 2010 from the Chanthaburi Mountain Range in Thailand (Figure 2B; see ref. [21]). None of the specimens studied belongs to an endangered or protected species. Specimens from Costa Rica were collected under the federal permission provided via the resolution number 123-2005-SINAC and exported under the federal permission number 014950 provided by the Gerencia Manejo y Uso Sostenible de RR NN – Ministerio del Ambiente y Energia to GM. Specimens of *Eoperipatus* sp. were obtained outside protected areas (hence no specific permission was required for these locations) and shipped under the export number 020-2481 7505 to Germany. The animals were housed in plastic boxes (200×100×65 mm) with perforated lids at 17°C (*Epiperipatus biolleyi*) or 22–24°C (*Principapillatus hitoyensis* **gen. et sp. nov.** and *Eoperipatus* sp.). The boxes were lined with a 2–3 cm layer of peat covered with damp paper tissues to retain moisture. All specimens were fed with recently killed crickets every 3–4 weeks. Peat and tissues were replaced two days after each feed.

### Morphological studies

A range of morphological methods was applied to numerous specimens of each species studied (Data S1: Table S1). Living specimens were photographed with a Nikon D70S camera under daylight. Specimens preserved in 70% ethanol were analysed and photographed with a stereomicroscope (Wild M10, Leica Microsystems, Wetzlar, Germany), equipped with a digital camera (PCO AG SensiCam, Kelheim, Germany). For scanning electron microscopy, specimens of both sexes and different ages, including dissected embryos, were fixed and preserved according to Read [2] with the following modifications: Specimens were killed by an exposure to chloroform vapour and placed in distilled water for 15 minutes, fixed in 4% formalin for one hour, rinsed in distilled water for 30 minutes and then dehydrated in an ethanol series. To avoid the separation of the cuticle from the body surface, mainly specimens that had recently moulted were used. After dehydration, they were dried in a critical point dryer (CPD 030 BAL-TEC AG, Balzers, Liechtenstein; and K850, Emitech Ltd., Kent, England), coated with gold in a SCD 050 Sputter Coater (BALZERS UNION, Balzers, Liechtenstein), and examined with the scanning electron microscopes Quanta 200 (FEI, Hillsboro, Oregon, USA) and EVO 50 (Carl Zeiss, Jena, Germany). Intra-specific character variation was assessed by comparing the data obtained from numerous specimens of different ages and both sexes. Terminology is used according to Reid [3] and Oliveira *et al.* [22] and additional terms are provided for novel structures described herein (Table 1).

**Table 1:** Description of morphological terms for Onychophora complementary to those provided by Reid [3] and Oliveira *et al.* [22].

Term	Description
Anal glands	Paired accessory genital glands in males, which are derivatives of nephridia of the limbless anal segment [65]; these glands open to the exterior either via a single orifice, as in *Eoperipatus* sp., or via two separate openings, as in most other peripatids [17], [59]
Antennal body	Antennal portion proximal to the antennal tip
Antennal sensory field	Field of spindle-shaped sensilla on the ventral surface of each antenna, present only in Peripatidae [3]; these structures were incorrectly referred to as “frontal organs” by Oliveira *et al.* [22]
Antennal tip (emended description)	Distal portion of antenna, which comprises the terminal button and a set of rings bearing an anterior row of chemoreceptors; the number of rings in the antennal tip is variable and it usually contains a few thin rings without chemoreceptors
Bean-shaped papilla	Bean-shaped structure situated dorsally in the distal portion of each leg in the neotropical species of Peripatidae [6]–[8]; the papilla lies in a pouch formed by tegumental folds
Chemoreceptor	Hemispherical sensory structure on the antennae covered by a thin cuticle and surrounded by a collar of scales
Crater-shaped papillae	Modified dermal papillae with a central depression surrounded by a collar of scales; present in both Peripatidae and Peripatopsidae (refs [4], [5]; present study); two types are recognised herein: the type I crater-shaped papillae are small, roundish and located on ventral plicae, whereas the type II crater-shaped papillae have elongated, oval bases and are situated in furrows between the plicae; the type II papillae are typically arranged in ventral and ventrolateral rows of six papillae between two subsequent leg pairs
Crural complex	Each crural complex consists of a pair of crural tubercles that are linked by a prominent dermal fold, forming a single unit covered with scales. The scales covering the anterior portion of the complex are modified and separated from the remaining scales by a clear border. So far, crural complexes have only been found in representatives of *Eoperipatus.*
Embryonic foot projections	Cuticular structures that occur in the distal leg portions in late embryos of the neotropical Peripatidae [9]–[11]; the foot projections can be smooth or barbed (refs [11], [12]; present study)
Frontal organ (emended description)	Slit-like organ formed by a tegumental ridge found proximally to the first antennal ring; the inner surface of the frontal organ is covered with numerous villus-like structures; note that this term was used incorrectly by Oliveira *et al.* [22] for antennal sensory fields with spindle-shaped sensilla
Interpedal structures	Fused or paired segmental structures covered with finely granulated cuticle; the interpedal structures occur in the furrow between the 5th and 6th plicae along ventral midline; their function is unknown
Lips	Tegumental folds, which surround the mouth opening; two circular rows of lips are found in Peripatidae and Peripatopsidae; their number and arrangement differ among onychophoran subgroups
Postoral pit	Unpaired transverse pit situated ventrally, posterior to the mouth from which it is separated by two plicae; the postoral pit is hard to see in contracted specimens; its function is unknown
Preventral organs	Segmental organs, which occur along ventral midline anterior to the ventral organs; preventral organs are smaller than ventral organs but show a similar structure; their function is unknown
Slime papilla	Modified limb situated laterally on the head; each slime papilla bears a slit-like opening of the slime gland; the opening is surrounded by denticle-like scales; the distal portion of slime papilla shows a variable pattern of dermal papillae; the term “oral papilla” is misleading and should not be used for this structure [3]
Spindle-shaped sensilla	Modified type I sensilla with enlarged, spindle-shaped basal pieces; they are found in condensed fields on the ventral surface near the antennal bases
Type I sensillum	Antennal sensillum composed of a prominent apical piece covered with scales and a bristle with a textured basis; present only in species of Peripatidae
Type II sensillum	Antennal sensillum composed of a bristle with a textured basis (apical piece absent)
Ventral fields of modified scales	Irregular fields of flattened scales (thus far only found in *Eoperipatus* sp.), which correspond in position with the interpedal structures in the neotropical species

### Molecular studies

Total DNA was extracted from fresh muscle tissue of a single specimen of each species using a NucleoSpin® Tissue Kit (Macherey-Nagel, Düren, Germany) according to the manufacturer's protocol. DNA was sheared with Covaris S2 Sonicator (Covaris Inc., Woburn, MA, USA). Starting at the blunt end repair step, a whole genome shotgun library was prepared following the multiplex protocol of Meyer & Kircher [23] with modifications for double indexing described in Kircher *et al.* [24]. This library was sequenced according to the manufacturer's instructions for single read multiplex experiments with 76 cycles paired-end on the Genome Analyzer IIx platform (v4 sequencing chemistry and v4 cluster generation kit, Illumina, San Diego, CA, USA). A second index read was performed according to Kircher *et al.* [24]. Raw sequences were analysed with IBIS 1.1.2 [25]. For highly accurate sample identification, sequences with falsely paired indexes were discarded. Paired-end reads from a single cluster were merged, if at least eleven base pairs were overlapping [26]. From these data, reads with more than five bases below a quality score of 15 and reads with low complexity were removed. The sequenced reads were assembled *de novo* using the CLC Genomics Workbench 4.7.2 (CLC bio, Aarhus, Denmark). *COI* and *12S rRNA* sequences were obtained from the whole genome shotgun libraries from the new species by BLAST searches [27]. Hereafter, specific primers were designed (COI_PH_F: 5′–CGGACGGAGTTAATAGTTATAGGG–3′, COI_PH_R: 5′–CATCCCAAAACCGGGCAAAATTA–3′, 12S_PH_F: 5′–GACAAGAATGACGGGCGATATGTAC–3′, 12S_PH_R: 5′–GCAAAATTCGTGCCAGCAGCAGC–3′). The *COI* sequence from *Eoperipatus* sp. was obtained from shotgun libraries as described above, whereas the *12S rRNA* sequence was amplified using the primers SR-J-14233 and SR-N-14588 from Simon *et al.* [28].

Additional cultured specimens of the new species (n = 21) were selected randomly and preserved in absolute ethanol. Genomic DNA was extracted using the NucleoSpin® Tissue Kit (Macherey-Nagel, Düren, Germany) following the manufacturer's protocol and fragments of the *COI* and *12S rRNA* genes were amplified and sequenced. The cycling reactions for *COI* (COI_PH_F/COI_PH_R) and *12S rRNA* (12S_PH_F/12A_PH_R) were set up with a first denaturation step for five minutes at 94°C, followed by 27 cycles including denaturation for 45 seconds at 94°C, annealing for 45 seconds at 56°C (*COI*) and at 54°C (*12S rRNA*), and elongation for one minute at 72°C. PCR products were purified using the NucleoSpin® Extract II Kit (Macherey-Nagel) following the manufacturer's protocol. The selected gene regions were sequenced in both directions using the Big DyeTM Terminator v.3.1 Cycle Sequencing Ready Reaction on an ABI PRISM 3100 sequencer (Applied Biosystems, Warrington, UK). Sequences were aligned using the online version of MAFFT [29], applying the FFT-NS-i strategy. The obtained sequences were placed in GenBank under specific accession numbers (Table 2).

**Table 2:** List of species used for phylogenetic analyses with corresponding GenBank accession numbers and references.

Species name	Accession number (*COI*)	Reference (*COI*)	Accession number (*12S rRNA*)	Reference (*12S rRNA*)
Peripatidae (ingroup):				
*Eoperipatus* sp.	JX569005	present study	JX568982	present study
*Epiperipatus acacioi*	HQ404902–05	Lacorte *et al.* [66]	HQ404920–23	Lacorte *et al.* [66]
*Epiperipatus adenocryptus* Oliveira *et al.*, 2011	HQ236113–14	Oliveira *et al.* [17]	HQ236139–40	Oliveira *et al.* [17]
*Epiperipatus biolleyi*	NC_009082	Podsialowiski *et al.* [67]	NC_009082	Podsialowiski *et al.* [67]
*Epiperipatus biolleyi*	HM600781	Rota-Stabelli *et al.* [68]	HM600781	Rota-Stabelli *et al.* [68]
*Epiperipatus diadenoproctus* Oliveira *et al.*, 2011	HQ236095–97	Oliveira *et al.* [17]	HQ236121–23	Oliveira *et al.* [17]
*Epiperipatus machadoi* (Oliveira & Wieloch, 2005)	HQ236089–90	Lacorte *et al.* [66]	HQ236115–16	Lacorte *et al.* [66]
*Epiperipatus paurognostus* Oliveira *et al.*, 2011	HQ236104–06	Oliveira *et al.* [17]	HQ236130–32	Oliveira *et al.* [17]
*Principapillatus hitoyensis* **gen. et sp. nov.**	JX568983–9004	present study	JX568960–81	present study
*Oroperipatus* sp.	NC01589	Segovia *et al.* [69]	NC015890	Segovia *et al.* [69]
*Peripatus solozanoi*	*	Morera-Brenes & Monge-Nájera [70]	–	–
Peripatopsidae (outgroup):				
*Euperipatoides rowelli* Reid, 1996	U62425	Gleeson *et al.* [18]	AF338016	Rockman *et al.* [19]
*Metaperipatus inae* Mayer, 2007	EF624055	Braband *et al.* [71]	EF624055	Braband *et al.* [71]
*Opisthopatus cinctipes* Purcell, 1899	NC014273	Braband *et al.* [72]	NC014273	Braband *et al.* [72]
*Peripatopsis moseleyi* (Wood-Mason, 1879)	EU855276	Daniels *et al.* [73]	EU855469	Daniels *et al.* [73]
*Phallocephale tallagandensis* Reid, 1996	U62407	Gleeson *et al.* [18]	AF338015	Rockman *et al.* [19]

* Sequences not found in GenBank but obtained from the original publication [70].

### Phylogenetic analyses

The Maximum-Likelihood (ML) inference method was applied for phylogenetic analyses using a combined dataset with *12S rRNA* sequences and either nucleotide or translated amino acid sequences of *COI* using the Invertebrate Mitochondrial Codon Table (code no. 5). Twenty-four additional sequences from eight species of Peripatidae (ingroup) and five species of Peripatopsidae (outgroup) were obtained from GenBank and from the literature (Table 2). Translated amino acid alignments were verified *a priori* using DAMBE [30]. The Maximum Likelihood analysis was conducted using RAxML 7.3.0 PTHREADS-SSE3 [31], selecting the substitution models GTR+G+I for nucleotides and MTART for amino acids. The latter was derived from PROTTEST3 [32] according to the Akaike information criterion [33]. Node support was calculated using 1,000 bootstrap pseudoreplicates [34].

### Karyology

Freshly dissected testes and seminal vesicles from specimens of *Principapillatus hitoyensis* **gen. et sp. nov.** and *Eoperipatus* sp. were used for cytogenetic analyses (Data S1: Table S1). The tissue was prepared according to Rowell *et al.* [35] with the following modifications. The tissue was dissected in a saline based on the onychophoran blood composition [36] at room temperature and placed for ten minutes in a hypotonic solution of water/saline (3∶1). Stained slides were mounted in Entellan (Merck, KGaA, Darmstadt, Germany) and analysed under a light microscope (Leitz DMR, Leica Microsystems). The images obtained from chromosome preparations were analysed using the freeware ImageJ 1.45s [37] with the plug-in “Levan” designed for chromosome classification [38]. Karyotype alignment was performed in Adobe (San Jose, CA, USA) Photoshop and Illustrator CS4. Information on the chromosome number in *Epiperipatus biolleyi* was obtained from the literature [16].

### Deposition of type specimens

Type specimens of the new species were deposited in the collections of the Museo de Zoología de la Universidad de Costa Rica (MZUCR), the Natural History Museum of London, England (BMNH), the Museum of Zoology [Senckenberg Natural History Collections], Dresden, Germany (SNSD), and the Universidade Federal de Minas Gerais, Brazil (UFMG).

### Nomenclatural acts

The electronic version of this document does not represent a published work according to the International Code of Zoological Nomenclature (ICZN) [39], and hence the nomenclatural acts contained in the electronic version are not available under that Code from the electronic edition. Therefore, a separate edition of this document was produced by a method that assures numerous identical and durable copies, and those copies were simultaneously obtainable (from the publication date noted on the first page of this article) for the purpose of providing a public and permanent scientific record, in accordance with Article 8.1 of the Code. The separate print-only edition is available on request from PLoS by sending a request to PLoS ONE, PLoS, 1160 Battery Street, Suite 100, San Francisco, CA 94111, USA along with a check for $10 (to cover printing and postage) payable to “PLoS”.

In addition, this published work and the nomenclatural acts it contains have been registered in ZooBank, the proposed online registration system for the ICZN. The ZooBank LSIDs (Life Science Identifiers) can be resolved and the associated information viewed through any standard web browser by appending the LSID to the prefix “http://zoobank.org/”. The LSID for this publication is: (urn:lsid:zoobank.org:pub:471AFB4F-A5B3-4002-AB87-5C2776E69402). The online version of this work is archived and available from the following digital repositories: PubMedCentral (www.pubmedcentral.nih.gov/), and LOCKSS (http://www.lockss.org/lockss/).

## Results

### Features shared by the three peripatid species

Scanning electron microscopy applied to specimens of different ages revealed a set of shared features among the three species of Peripatidae studied: (i) *Eoperipatus* sp. from Thailand, (ii) *Epiperipatus biolleyi* from Costa Rica, and (iii) the new species and genus from Costa Rica, which is described herein as *Principapillatus hitoyensis* **gen. et sp. nov.** (see the taxonomic description below).

**Figure 3:**
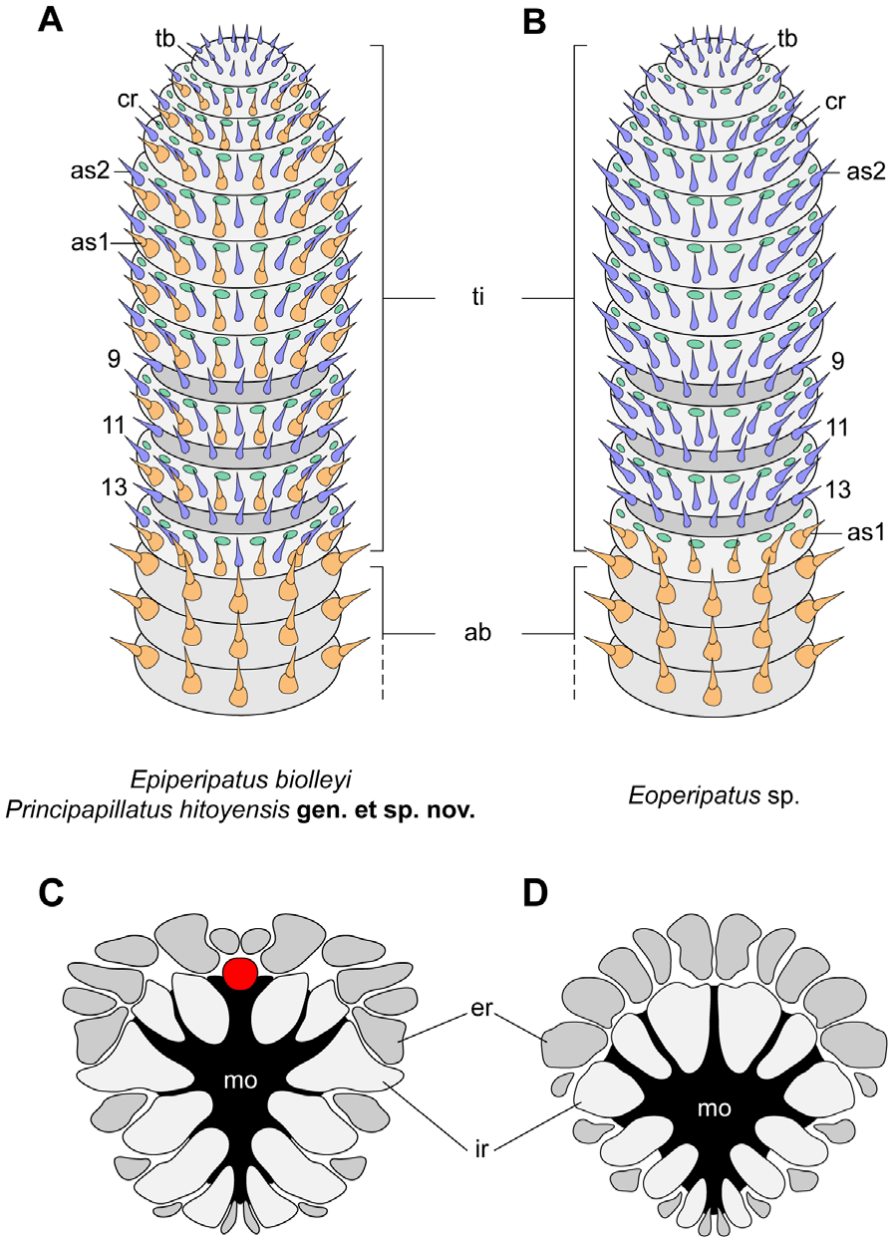
Diagrams of antennal tips and mouth lips in the three species studied. (A, B) Distribution of chemoreceptors (green) and type I (light-brown) and type II sensilla (blue) on the antennal tip. (A) *Principapillatus hitoyensis* **gen. et sp. nov.** and *Epiperipatus biolleyi*. (B) *Eoperipatus* sp. Reduced rings are highlighted in dark-grey and numbered. Note that type I sensilla are absent from the antennal tip in *Eoperipatus* sp. (C, D) Arrangement of lips surrounding the mouth opening. (C) *Principapillatus hitoyensis* **gen. et sp. nov.** and *Epiperipatus biolleyi*. (D) *Eoperipatus* sp. The external row is indicated in dark-grey. Unpaired lip (in the neotropical species only) is highlighted in red. Abbreviations: ab, antennal body; as1, type I sensillum; as2, type II sensillum; cr, chemoreceptor; er, external row; ir, internal row; mo, mouth; tb, terminal button; ti, antennal tip.

**Figure 4:**
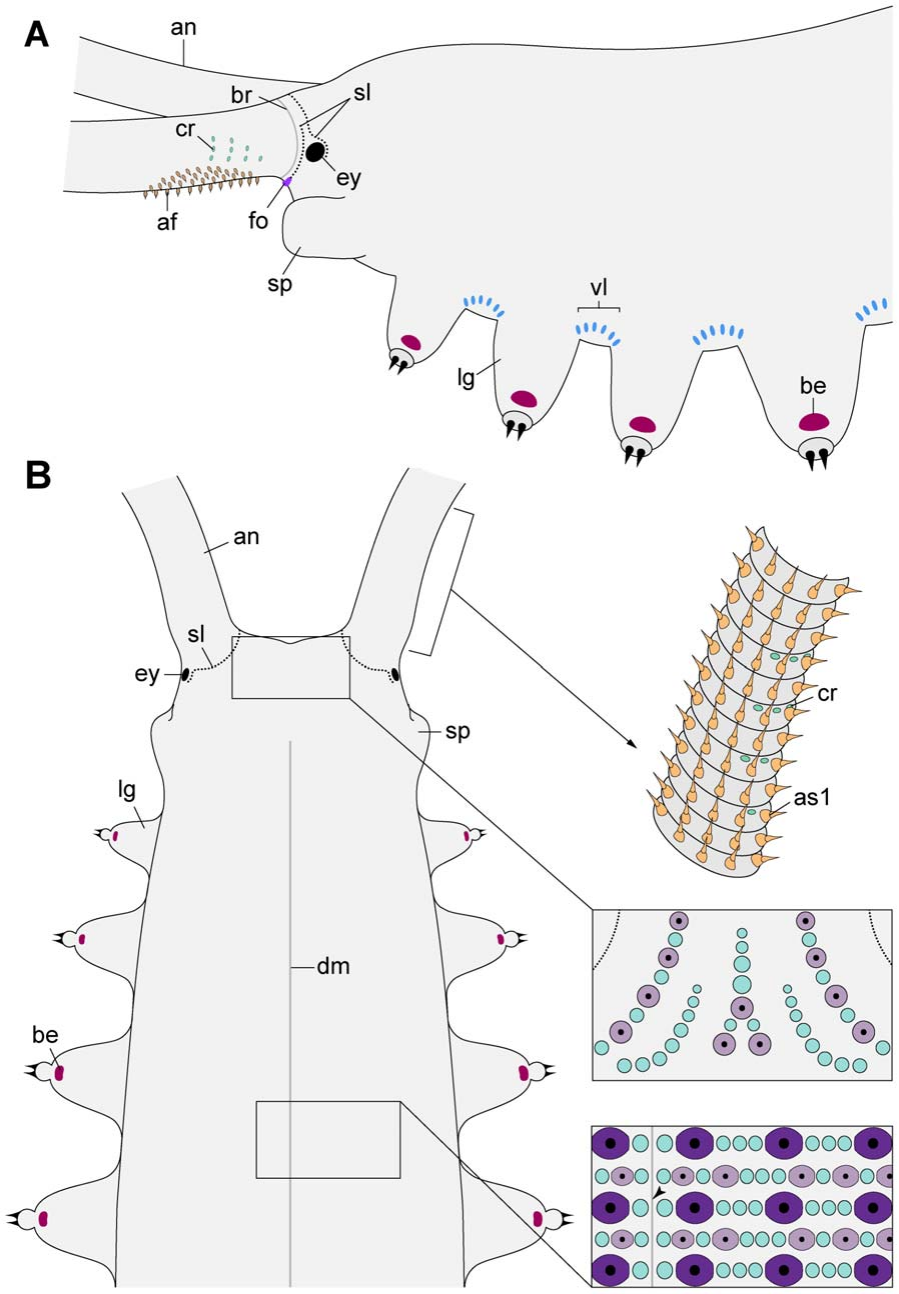
Diagrams of lateral and dorsal structures in *Principapillatus hitoyensis* gen. et sp. nov. (A) Anterior body portion in lateral view. Note the fields of spindle-shaped sensilla and ventrolateral chemoreceptors on the antenna. (B) Anterior end in dorsal view. Insets show details of the antennal base, the head pattern and the arrangement of dermal papillae in the dorsal integument (arrowhead points to the dorsomedian furrow). Abbreviations: an, antenna; af, antennal sensory field with spindle-shaped sensilla; be, bean-shaped papilla; br, basal-most antennal ring (indicated by a thin dark-grey line); cr, chemoreceptor; dm, dorsomedian furrow (thick dark-grey line); ey, eye; fo, frontal organ; lg, leg; as1, type I sensillum; sl, spiralling plica (indicated by a dotted line); sp, slime papilla; vl, ventrolateral row of type II crater-shaped papillae.

**Figure 5:**
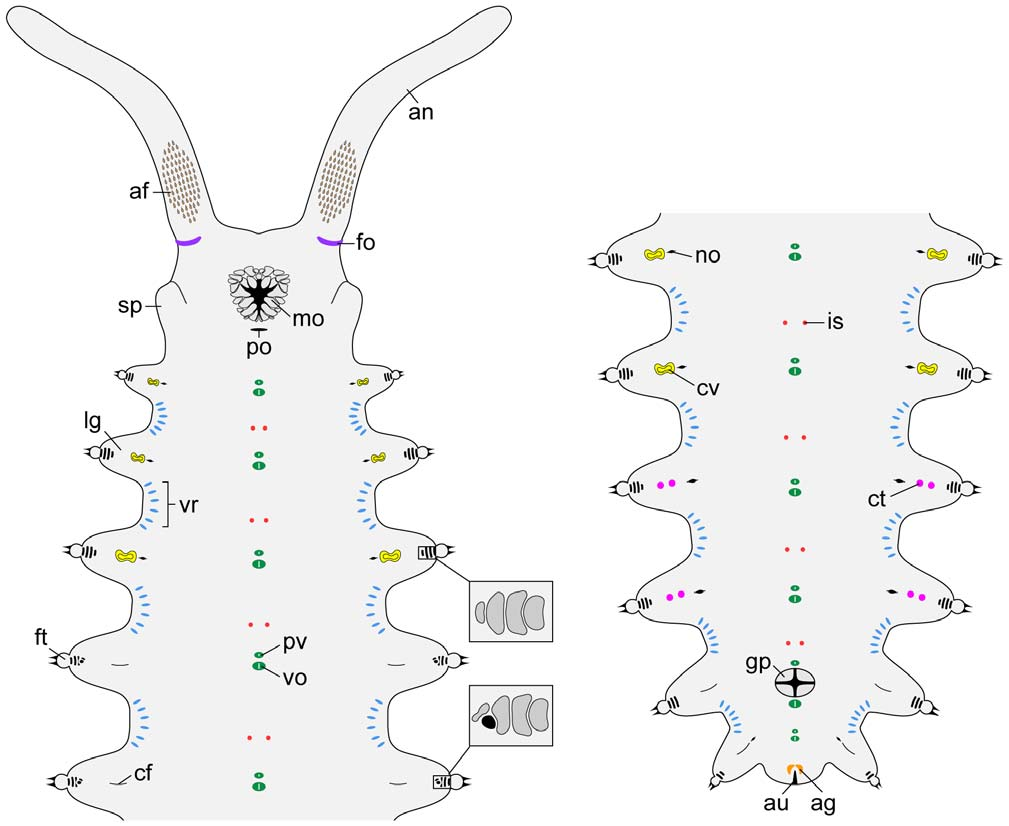
Diagrams of ventral structures in *Principapillatus hitoyensis* gen. et sp. nov. Anterior (left) and posterior (right) body portions showing the position of various morphological structures analysed in the present study. The diagram of the posterior end on the right represents a male. Note the position of the genital pad between preventral and ventral organs of the genital segment. Insets show details of spinous pads and position of nephridial tubercle (black) in the fifth leg pair. Abbreviations: af, antennal sensory field with spindle-shaped sensilla; ag, anal gland opening; an, antenna; au, anus; cf, coxal furrow; ct, crural tubercle; cv, coxal vesicle; fo, frontal organ; ft, foot; gp, genital pad; is, interpedal structure; lg, leg; mo, mouth; no, nephridial opening; po, postoral pit; pv, preventral organ; sp, slime papilla; vo, ventral organ; vr, ventral row of type II crater-shaped papillae.

**Figure 6:**
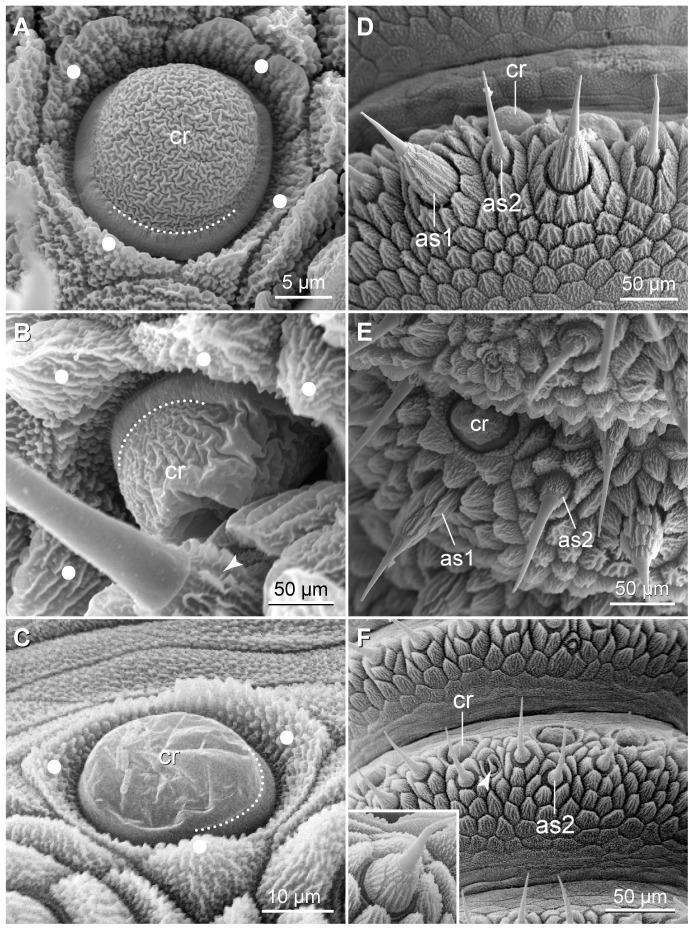
Scanning electron micrographs of sensory structures on the antennal tip. (A–C) Chemoreceptors. (A) *Principapillatus hitoyensis* **gen. et sp. nov.** (B) *Epiperipatus biolleyi*. (C) *Eoperipatus* sp. Note the scales surrounding the structure (white dots) and the smooth peripheral ring (white dotted line). The arrowhead in B points to the textured basis of a type II sensillum. (D–F), Antennal type I sensilla and type II sensilla. (D) *Principapillatus hitoyensis* **gen. et sp. nov.** (E) *Epiperipatus biolleyi*. *Eoperipatus* sp. (F). Anterior is up. Note that type I sensilla are missing on the antennal tip in *Eoperipatus* sp. Inset in F shows detail of a type I sensillum from the antennal body. Note that underdeveloped type II sensilla (arrowhead in F) are found in all three species studied. Abbreviations: as1, type I sensillum; as2, type II sensillum; cr, chemoreceptor.

**Figure 7:**
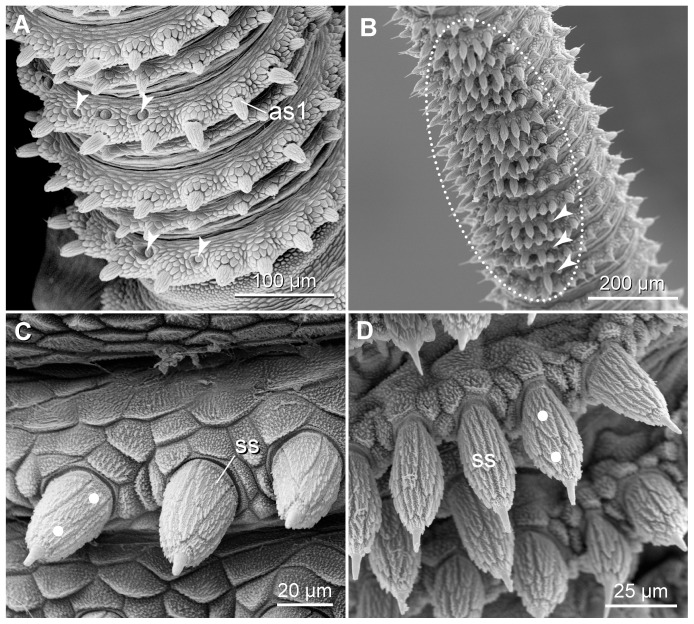
Scanning electron micrographs of sensory structures found on the antennal body. Anterior is up in all images. (A) Dorsolateral view of the proximal region of an antenna in *Eoperipatus* sp. Lateral is left. Note a few chemoreceptors (arrowheads) situated on alternate rings. (B) Sensory field (dotted line) with spindle-shaped sensilla on the ventral surface of an antenna in *Principapillatus hitoyensis* **gen. et sp. nov.** Arrowheads point to the semi-rings with spindle-shaped sensilla. (C, D) Detail of spindle-shaped sensilla. (C) *Eoperipatus* sp. (D) *Principapillatus hitoyensis* **gen. et sp. nov.** White dots indicate the scale ranks in the apical piece. Abbreviations: as1, type I sensillum; ss, spindle-shaped sensillum.

The antennae of the three species show a variable number of rings in the antennal body and 14 rings in the antennal tip (including the terminal button), of which the 9^th^, 11^th^ and 13^th^ rings are thinner than the others (Figure 3A, B). Four types of sensory structures are found on the entire antennae in the species studied: one type of putative chemoreceptor (“sensory bulbs” according to ref. [40]) and three types of putative mechanoreceptors [40], [41], including type I sensilla, type II sensilla and spindle-shaped sensilla (Figures 3A, B, 4A, B, 5, 6A–F). The composition of chemoreceptors is similar among the species. They are hemispherical structures covered by a thin cuticle, which appears wrinkled in the central region but forms a smooth ring in the periphery (Figure 6A–C). Each chemoreceptor is surrounded by two to four scales (Figure 6A). Although most chemoreceptors occur on the antennal tip, where they lie in an anterior row on each ring (Figures 3A, B, 6D–F), a few additional chemoreceptors are found dorsolaterally on alternate rings in the proximal antennal region (Figures 4A, B, 7A).

The three types of antennal mechanoreceptors show a different structure. The type I sensilla are composed of a prominent apical piece covered with scales and a sensory bristle with a textured basis (Figure 6B), while the type II sensilla do not have an apical piece but consist of a long, needle-shaped bristle with a textured basis (Figures 3A, B, 6B, D–F). The third type, the spindle-shaped sensilla, are modified type I sensilla since they show an enlarged, spindle-shaped apical piece and a short, thorn-shaped bristle (Figure 7B–D). The spindle-shaped sensilla only occur in sensory fields located on the proximoventral surface of each antenna (Figures 4A, 5, 7B). Notably, the terminal button ( = first antennal, discoid “ring”) and the thin reduced rings of the antennal tip exclusively show type II sensilla (Figure 3A, B).

**Figure 8:**
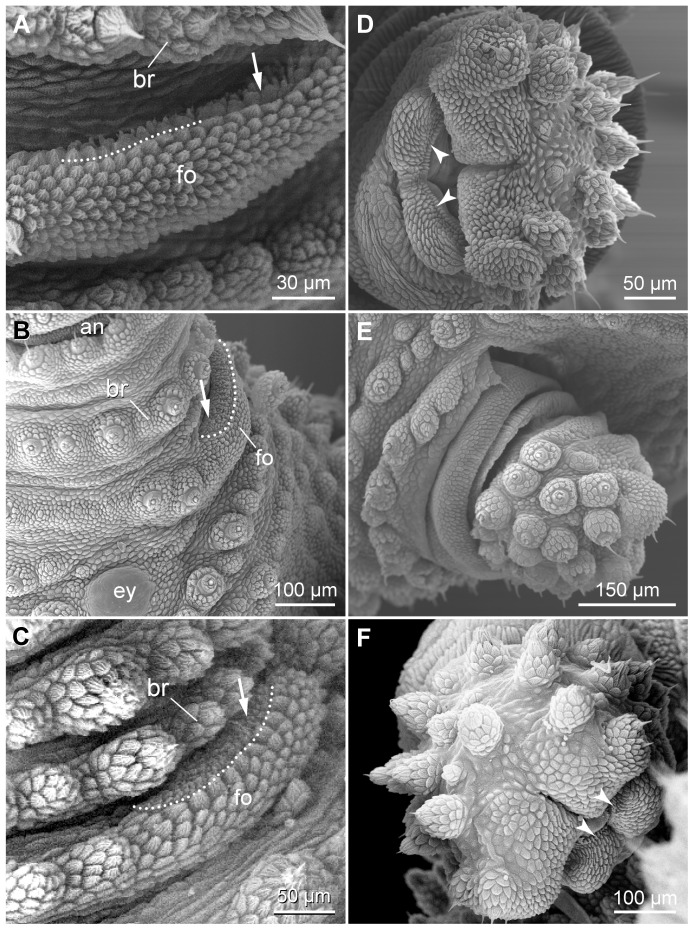
Scanning electron micrographs of frontal organs and slime papillae. (A–C) Details of frontal organs. (A) *Principapillatus hitoyensis* **gen. et sp. nov.** (B) *Epiperipatus biolleyi.* (C) *Eoperipatus* sp. Anterior is up. Dotted line demarcates the internal region with villus-like structures (arrow). (D–F) Details of slime papillae. (D) *Principapillatus hitoyensis* **gen. et sp. nov.** (E) *Epiperipatus biolleyi*. (F) *Eoperipatus* sp. Note the denticle-like scales (arrowheads) surrounding the opening of the slime gland in D and F. Abbreviations: an, antenna; br, basal-most antennal ring; fo, frontal organ.

Additional cephalic sensory structures include the frontal organs, which are situated anteriorly on the head (Figures 4A, 5, 8A–C). The frontal organs are slit-like structures formed by a dermal ridge, which is part of the spiralling plica associated with the eye, i.e., the plica that follows posteriorly the basal-most antennal ring (Figures 4A, 8A–C). The external surface of the ridge lacks dermal papillae but is covered by scales whereas its internal surface shows a modified cuticle with numerous villus-like structures (Figure 8A–C). The slit of the frontal organ appears narrow in some specimens but is widely open in others.

The structure of the slime papillae is similar in the three species studied (Figure 8D–F). The slit-like opening of the slime gland on the tip of the slime papilla is shifted medially and delineated by a row of regular, denticle-like scales (Figure 8D, F). Despite these similarities, the set of dermal papillae found distally on the slime papillae differs intraspecifically and even between each side of the same specimen.

**Figure 9:**
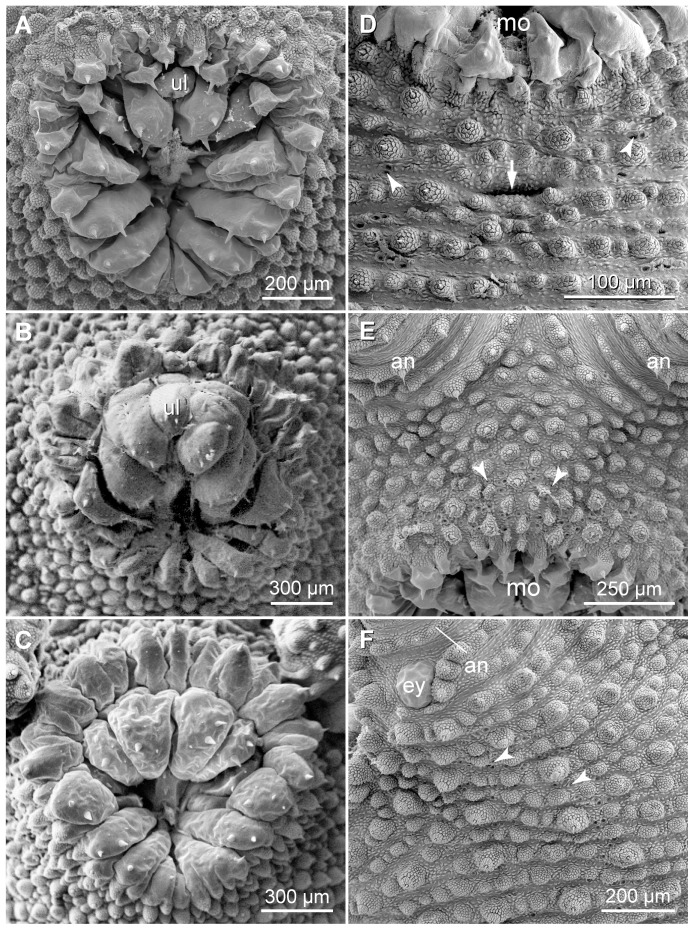
Scanning electron micrographs showing the arrangement of oral lips and fields of tracheal openings. Anterior is up in all images. (A–C) Details of oral lips. (A) *Principapillatus hitoyensis* **gen. et sp. nov.** (B) *Epiperipatus biolleyi*. (C) *Eoperipatus* sp. Note the anterior unpaired lip in the two neotropical species (images A and B). (D–F) Condensed fields of tracheal openings, here exemplified only for *Principapillatus hitoyensis* **gen. et sp. nov.** Arrowheads point to single tracheal openings ( = tracheal atria). (D) Ventral body surface behind the mouth. Arrow points to the postoral pit. (E) Head in frontal view showing a tracheal field in front of the mouth. (F) Dorsolateral head region. Note a tracheal field behind the eye. Abbreviations: an, antenna; ey, eye; mo, mouth; ul, anterior unpaired lip.

The mouth opening of each species is surrounded by an internal and an external row of lips (Figures 3C, D, 9A–C). The lips of the internal row are larger than those of the external row. The jaws are situated within the mouth cavity and are composed of a pair of inner and outer blades, each showing a primary tooth and a variable number of accessory teeth. The inner jaw blades have, in addition, a diastema and a row of denticles.

Condensed fields of tracheal openings ( = tracheal atria) occur posterior to each eye and anterior and posterior to the mouth opening (Figure 9D–F). Furthermore, a medial transverse pit ( = postoral pit) occurs ventrally behind the mouth (Figures 5, 9D). The postoral pit is not seen in scanning electron micrographs of contracted specimens.

**Figure 10:**
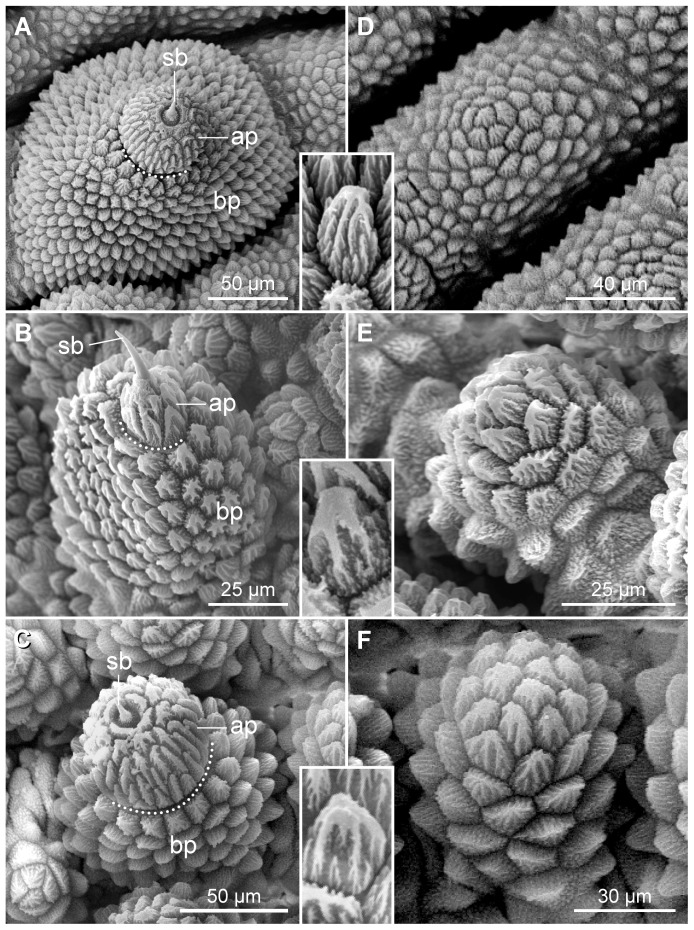
Scanning electron micrographs of dermal papillae. Insets show details of single scales from the basal piece in each species. (A–C) Primary papillae. (A) *Principapillatus hitoyensis* **gen. et sp. nov.** (B) *Epiperipatus biolleyi*. (C) *Eoperipatus* sp. The dotted lines indicate constrictions separating the basal and apical pieces of the primary papillae. (D–F) Accessory papillae. (D) *Principapillatus hitoyensis* **gen. et sp. nov.** (E) *Epiperipatus biolleyi*. (F) *Eoperipatus* sp. Note the different size, shape and composition of dermal papillae among the species. Abbreviations: ap, apical piece; bp, basal piece; sb, sensory bristle.

The dermal papillae in the three species show roundish bases and are situated on plicae of which there are twelve per segment (Figure 10A–F). Only seven of these plicae pass between adjacent leg pairs to the ventral body surface. Each primary papilla is subdivided by a constriction into an apical piece bearing a sensory bristle and a basal piece, which is larger than the apical piece (Figure 10A–C). In contrast, the accessory papillae lack apical pieces and sensory bristles (Figure 10D–F). The primary and accessory papillae vary in size and are more slender in the lateral and anal body regions than those on the dorsal body surface. Primary and accessory papillae are also present on the ventral body surface, but they are smaller in size and show an irregular shape as compared to those on the dorsal surface.

**Figure 11:**
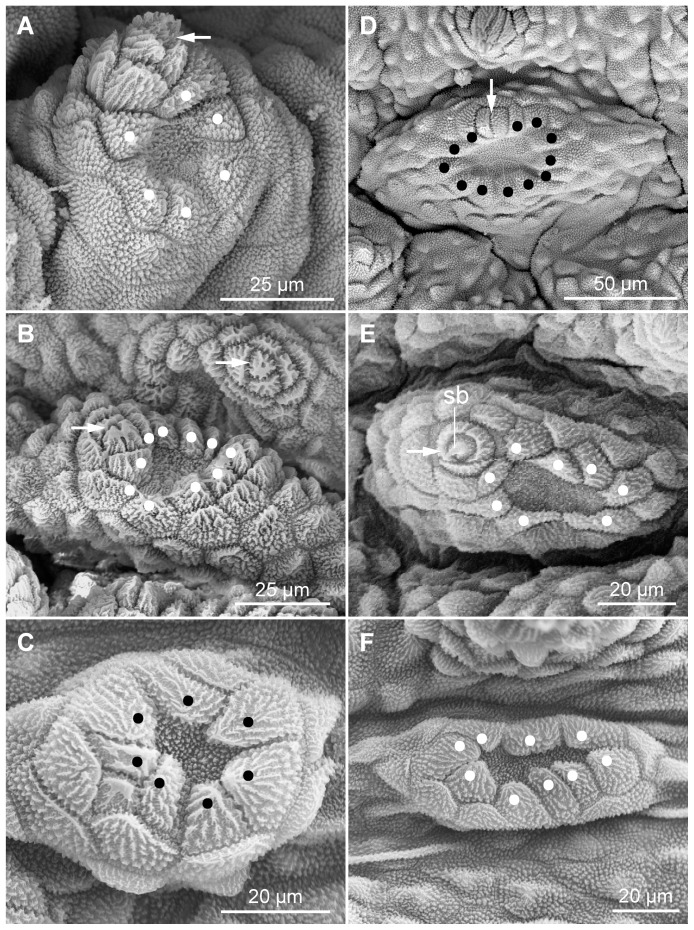
Scanning electron micrographs of the two types of crater-shaped papillae. Anterior is up and lateral is left in all images. White and black dots indicate the scales in the apical collar. (A–C) Type I crater-shaped papillae. (A) *Principapillatus hitoyensis* **gen. et sp. nov.** (B) *Epiperipatus biolleyi*. (C) *Eoperipatus* sp. (D–F) Type II crater-shaped papillae. (D) *Principapillatus hitoyensis* **gen. et sp. nov.** (E) *Epiperipatus biolleyi*. (F) *Eoperipatus* sp. Note the occurrence of a rudimentary apical piece in the crater-shaped papillae of the two neotropical species (arrows in A, B, D, E). Abbreviation: sb, sensory bristle.

**Figure 12:**
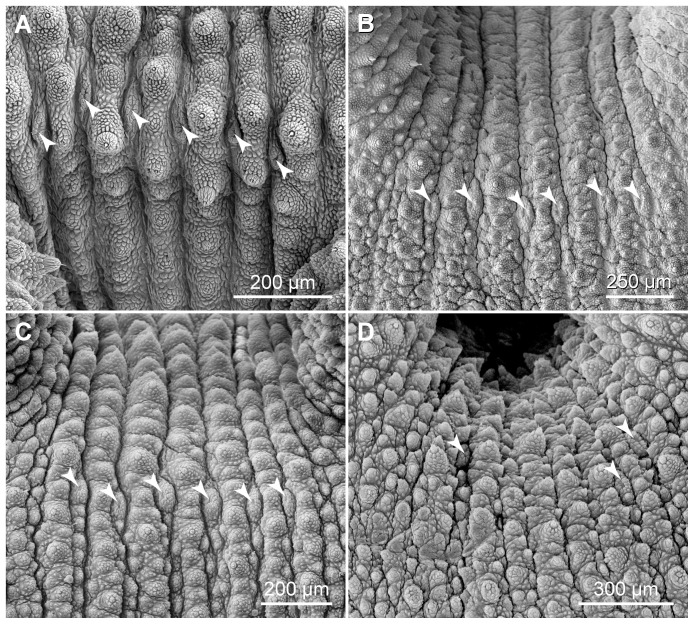
Scanning electron micrographs showing the arrangement of the type II crater-shaped papillae. Anterior is right in all images. Arrowheads point to single crater-shaped papillae. (A) Ventrolateral row of type II crater-shaped papillae in *Principapillatus hitoyensis* **gen. et sp. nov.** (B–D) Ventral row of type II crater-shaped papillae. (B) *Principapillatus hitoyensis* **gen. et sp. nov.** (C) *Epiperipatus biolleyi*. (D) *Eoperipatus* sp. Note the regular rows of six crater-shaped papillae in the two neotropical species (images A–C) in contrast to the less regular arrangement of these papillae in *Eoperipatus* sp. (image D).

Notably, highly modified, crater-shaped papillae are found on the ventral and ventrolateral body surface in the three species studied (Figure 11A–F). These papillae are characterised by a central depression with a granulated cuticle, which is surrounded by a collar of scales (Figure 11A–F). Two types of crater-shaped papillae can be distinguished according to their structure and position. The type I crater-shaped papillae are relatively small, roundish and scattered on the plicae of the ventral integument (Figure 11A–C), whereas the type II papillae have an elongated, oval basis and are located in furrows between the seven plicae passing to the ventral body surface (Figure 11D–F). There are two rows of the type II papillae on each body side, a ventrolateral and a ventral row (Figure 12A–D).

**Figure 13:**
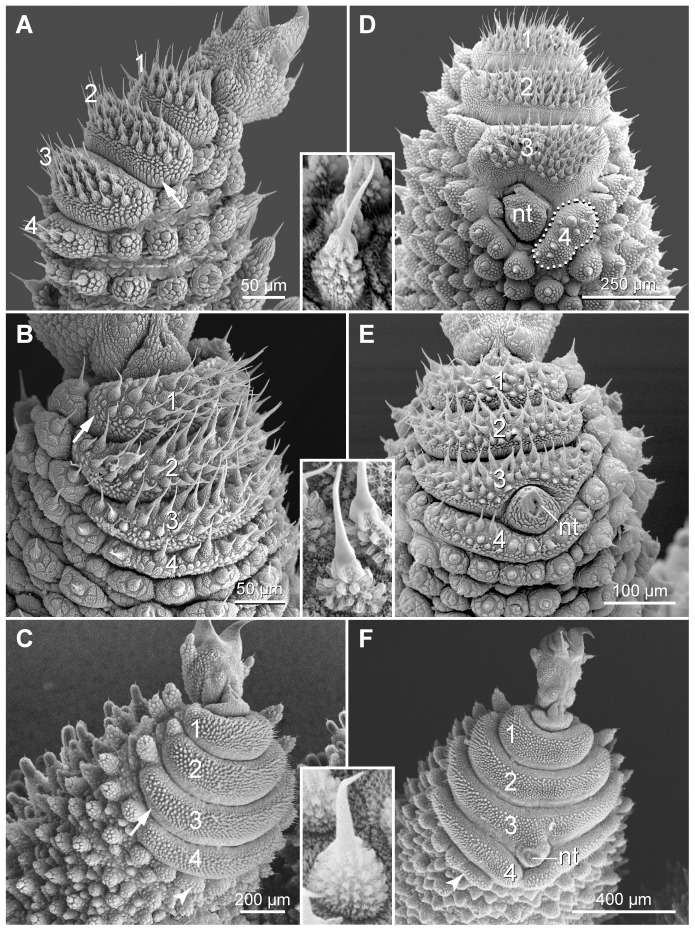
Scanning electron micrographs showing characteristics of the spinous pads. Anterior is right in all images. Insets show details of bristles from the spinous pads of each species. (A–C) Details of legs from the midbody region. (A) *Principapillatus hitoyensis* **gen. et sp. nov.** (B) *Epiperipatus biolleyi*. (C) *Eoperipatus* sp. Arrows point to a bristle-less region of the spinous pad. (D–F) Details of legs showing the position of nephridial tubercle in the fourth and fifth leg pairs. (D) *Principapillatus hitoyensis* **gen. et sp. nov.** (E) *Epiperipatus biolleyi*. (F) *Eoperipatus* sp. Note the interspecific differences in size and shape of the spinous pads (numbered). Note also similar position of the nephridial tubercle in the three species studied. Arrowheads in C and F point to the rudimentary fifth spinous pad. Abbreviation: nt, nephridial tubercle.

Most legs in the three species studied have four separate spinous pads the number of which is lower in the anterior-most and two posterior-most leg pairs (Figures 5, 13A–F). The spines are present only on the flattened central surface, whereas the periphery of each spinous pad is covered with flat, elongated scales (Figure 13A). Each spine shows a structured basis, which differs among the three species (Figure 13: insets). The nephridial tubercle is located between the third and fourth spinous pads in the fourth and fifth leg pairs (Figure 13D–F). In these leg pairs, no coxal vesicles are found, whereas a single eversible coxal vesicle occurs in each coxal furrow of the remaining leg pairs (Figure 5; Data S1: Figure S1A, B). When everted, the surface of coxal vesicles is smooth and appears white under a stereomicroscope (Data S1: Figure S1A).

**Figure 14:**
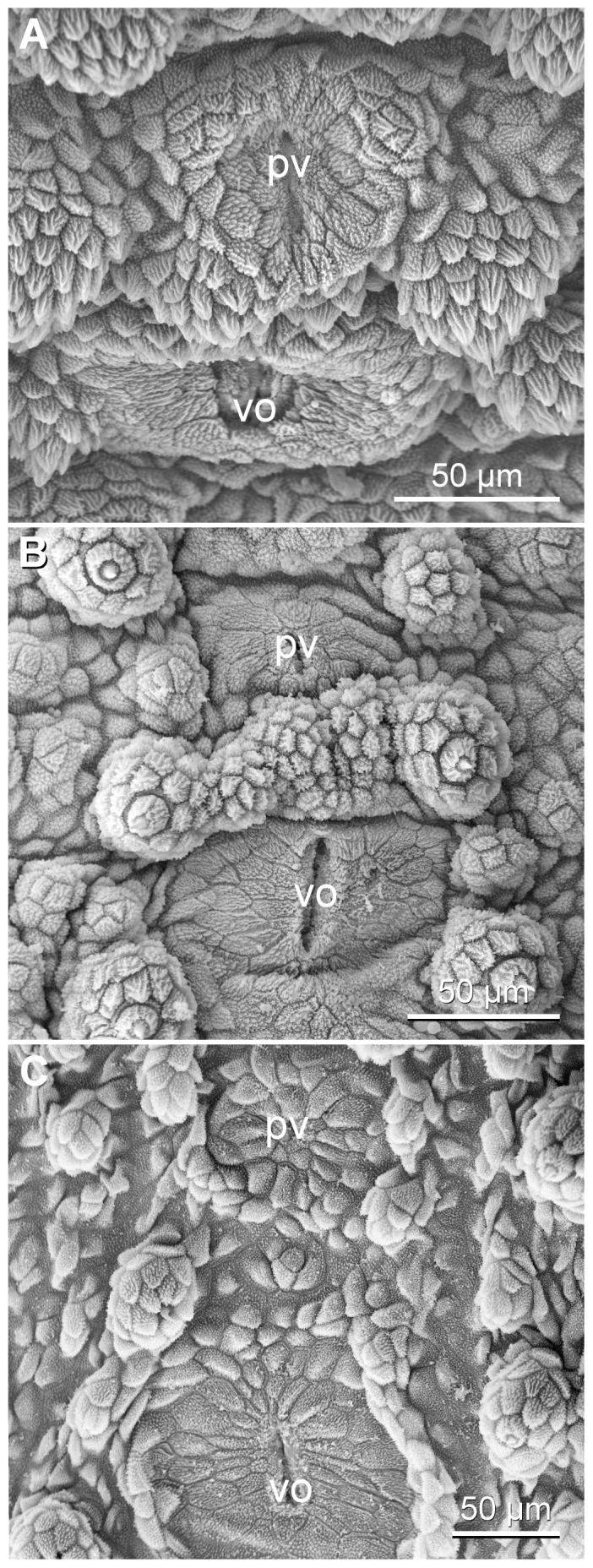
Scanning electron micrographs of ventral and preventral organs. Anterior is up in all images. (A) *Principapillatus hitoyensis* **gen. et sp. nov.** (B) *Epiperipatus biolleyi*. (C) *Eoperipatus* sp. Abbreviations: pv, preventral organ; vo, ventral organ.

**Figure 15:**
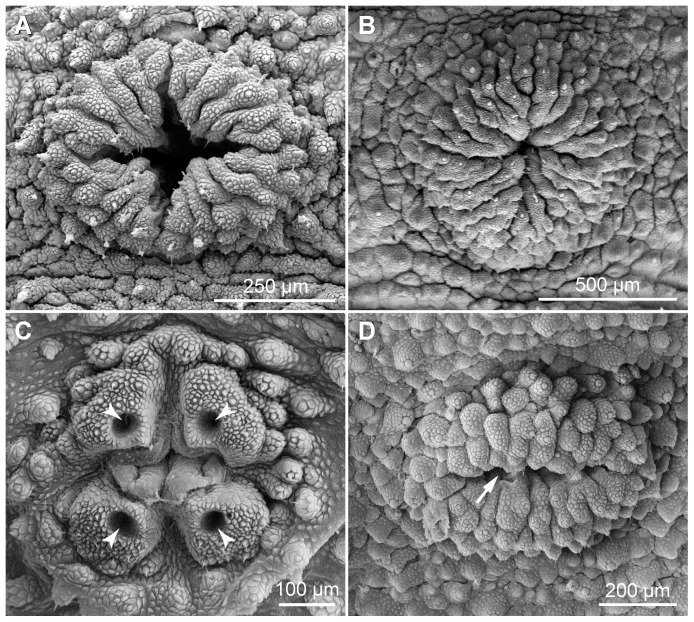
Scanning electron micrographs of the genital pads. Anterior is up in all images. (A) Male genital pad in *Principapillatus hitoyensis* **gen. et sp. nov.** (B) Female genital pad in *Principapillatus hitoyensis* **gen. et sp. nov.** (C) Male genital pad in *Eoperipatus* sp. Note the four circular pits (arrowheads). (D) Female genital pad in *Eoperipatus* sp. Note the slit-like appearance of the genital opening (arrow).

**Figure 16:**
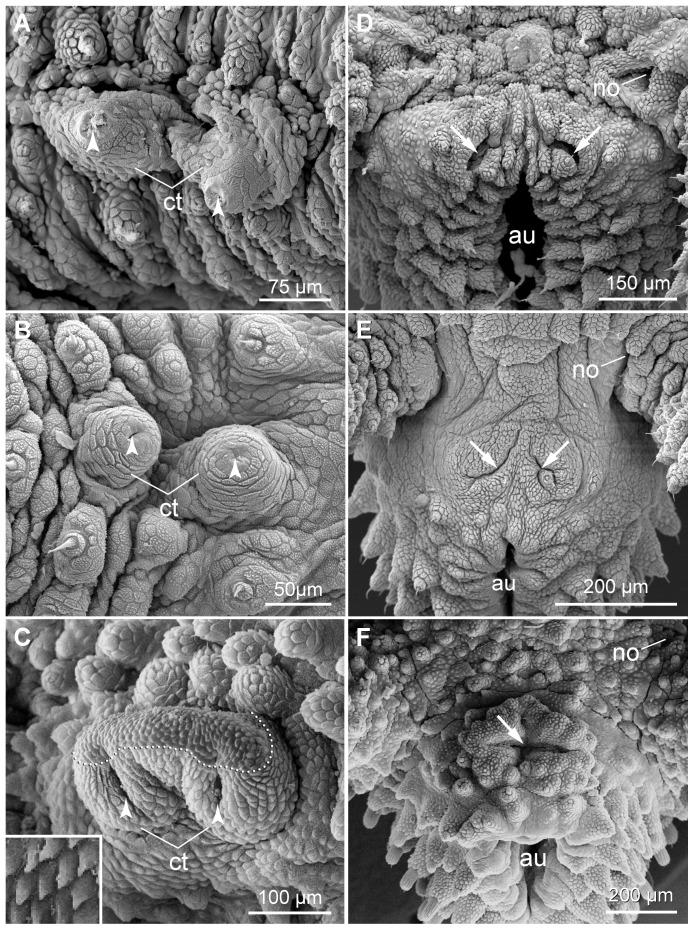
Scanning electron micrographs of male reproductive structures. Anterior is up in all images. (A–C) Crural tubercles. (A) *Principapillatus hitoyensis* **gen. et sp. nov.** (B) *Epiperipatus biolleyi*. (C) *Eoperipatus* sp. Note that the crural tubercles are separate and show smooth apices in the neotropical species (images A and B), whereas they are linked by a dermal fold and completely covered with scales in *Eoperipatus* sp. (image C). Arrowheads point to the openings of crural glands. Dotted line in C demarcates the anterior region of the crural complex in *Eoperipatus* sp., which is covered with modified scales (details of these modified scales are shown in the inset). (D–F) Details of body region associated with anal gland openings (arrows). (D) *Principapillatus hitoyensis* **gen. et sp. nov.** (E) *Epiperipatus biolleyi*. (F) *Eoperipatus* sp. Note that two separate, slit-like openings occur in the two neotropical species (images D and E), whereas there is only a single median, cruciform opening situated on a specialised pad in *Eoperipatus* sp. (image F). Abbreviations: au, anus; ct, crural tubercles; no, nephridial opening.

Paired ventral and smaller preventral organs are found along the ventral midline between each leg pair (Figures 5, 14A–C). In most segments, these organs lie next to each other, except for the penultimate leg-bearing segment in which the genital pad is situated between them (Figure 5). The genital opening appears cruciform in males but slit-like in females of the three species (Figures 5, 15A–D). Crural glands are present only in two pregenital segments in males. In these segments, two openings of crural glands occur on the ventral surface of each leg (Figures 5, 16A–C). The anal glands are also found only in males. They open to the exterior via a paired or unpaired opening situated ventrally in the posterior-most, limbless body region referred to as the anal cone (Figures 5, 16D–F).

### Features shared by the two neotropical species

Our data revealed a set of characters found only in *Epiperipatus biolleyi* and *Principapillatus hitoyensis* **gen. et sp. nov.** but not in *Eoperipatus* sp.. One of these characters is the distribution of type I sensilla along the antennae, which are present on the antennal tip only in the two neotropical species but are restricted to the antennal body and the proximal-most ring of the antennal tip in *Eoperipatus* sp. (Figures 3A, B, 6D–F). Furthermore, the internal row of lips surrounding the mouth opening bears an unpaired anterior lip only in the neotropical species (Figures 3C, D, 8A–C). There are also structural differences in the dorsal integument among the species. Typically, the integument of the neotropical species shows two incomplete plicae in each leg-bearing region, which anastomose with each other dorsally and do not extend laterally. The position of the incomplete plicae may vary between adjacent segments of a single specimen. In contrast to the neotropical species, the dorsal integument of *Eoperipatus* sp. does not show incomplete plicae, although some of them anastomose laterally above each leg.

The dorsal primary papillae of the two neotropical species bear a sensory bristle in the middle of each apical piece (Figure 10A, B). In contrast, the apical piece is asymmetric in *Eoperipatus* sp., as it shows a lower posterior number of scale ranks and the sensory bristle is shifted posteriorly (Figure 10C). The structure of both types of crater-shaped papillae also differs among the species, as they possess a rudimentary apical piece in the neotropical species, whereas the apical piece is missing in *Eoperipatus* sp. (Figure 11A–F). Some of these rudimentary apical pieces in *Epiperipatus biolleyi* and *Principapillatus hitoyensis* **gen. et sp. nov.** bear a well-developed sensory bristle, while others appear as rudimentary bumps consisting of only a few scales (Figure 11A–D). Furthermore, the type II crater-shaped papillae are arranged in regular rows of exactly six papillae in the neotropical species, whereas their arrangement is less regular and their number lower in *Eoperipatus* sp. (Figure 12A–D).

**Figure 17:**
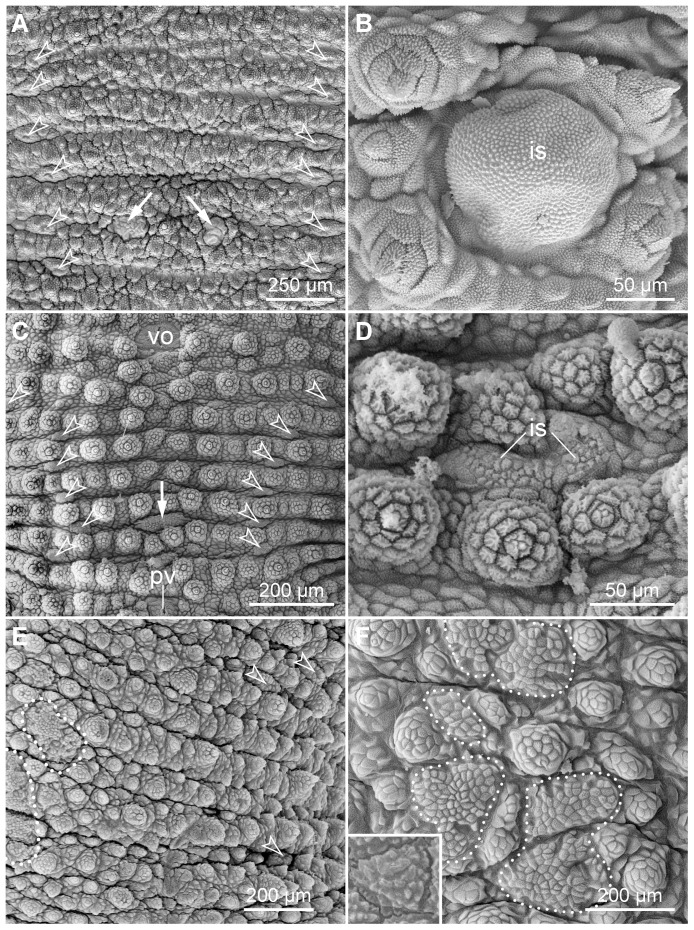
Scanning electron micrographs of the interpedal structures and ventral fields with modified scales. Anterior is up in all images. Arrowheads point to the type II crater-shaped papillae. (A–D), Interpedal structures. (A, B) *Principapillatus hitoyensis* **gen. et sp. nov.** (C, D) *Epiperipatus biolleyi*. Note that the interpedal structures (arrows) lie in a similar position in both species, but they are separate and covered with a finely granulated cuticle in *Principapillatus hitoyensis* **gen. et sp. nov.** (images A and B), whereas they are fused and covered with modified scales of different size in *Epiperipatus biolleyi* (images C and D). (E, F) Ventral fields of modified scales (dotted lines) in *Eoperipatus* sp. Note the irregular shape of the field. Inset in F shows details of a single modified scale from the ventral field. Abbreviation: is, interpedal structure; pv, preventral organ; vo, ventral organ.

Notably, there are segmental structures in the ventral integument of the two neotropical species, which we refer to as interpedal structures (Figures 5, 17A–D). These structures are situated in furrows between the fifth and sixth plicae passing to the ventral surface between subsequent leg pairs (Figure 5). The interpedal structures are paired in *Principapillatus hitoyensis* **gen. et sp. nov.**, whereas they are fused along the ventral midline in *Epiperipatus biolleyi*. These structures occur in all but the last leg-bearing segments in these species, while they are missing completely in *Eoperipatus* sp., which instead shows wide, irregular fields of flattened scales in the corresponding regions of the ventral integument (Figure 17E, F).

**Figure 18:**
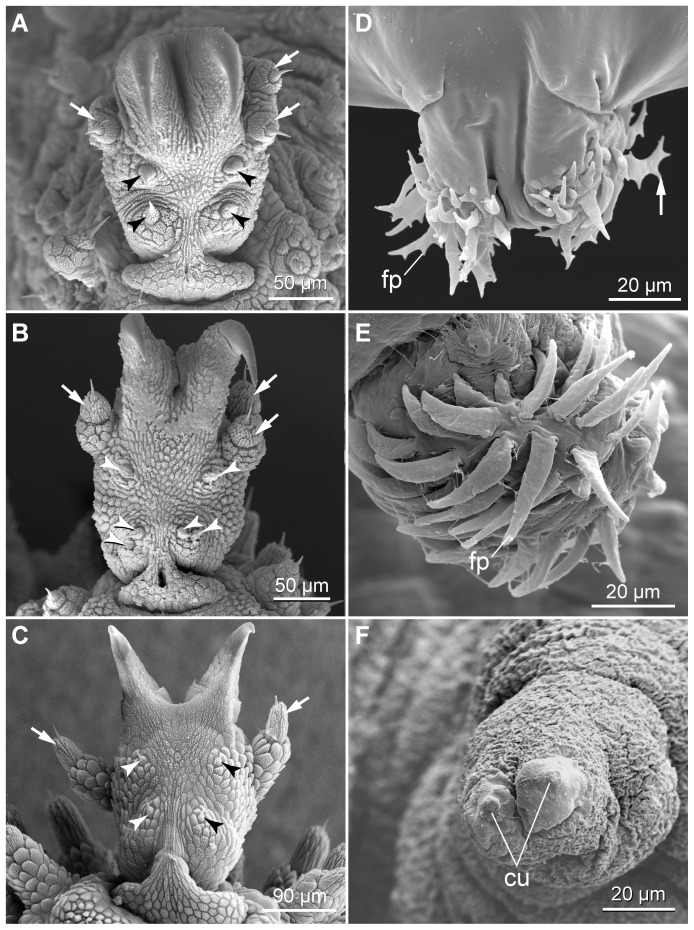
Scanning electron micrographs of distal leg portions. (A–C) Details of feet in adults. (A) *Principapillatus hitoyensis* **gen. et sp. nov.** (B) *Epiperipatus biolleyi*. (C) *Eoperipatus* sp. Anterior is right. Arrows indicate two anterior and one posterior distal foot papillae in the neotropical species (images A and B) and one anterior and one posterior foot papilla in *Eoperipatus* sp. (image C). Arrowheads point to bristles in the distal and proximal setiform ridges. (D–F) Details of feet in embryos at an advanced developmental stage (nearly fully developed, pigmented embryos). (D) *Principapillatus hitoyensis* **gen. et sp. nov.** (E) *Epiperipatus biolleyi*. (F) *Eoperipatus* sp. Note the occurrence of embryonic foot projections in the neotropical species (images D and E). The foot projections are barbed in embryos of *Principapillatus hitoyensis* **gen. et sp. nov.** (arrows in D), whereas they are smooth in *Epiperipatus biolleyi* (image E). Note also that the embryonic foot projections are missing in the embryo of *Eoperipatus* sp. (image F). Abbreviations: cu, embryonic cuticle covering the claws; fp, embryonic foot projections.

**Figure 19:**
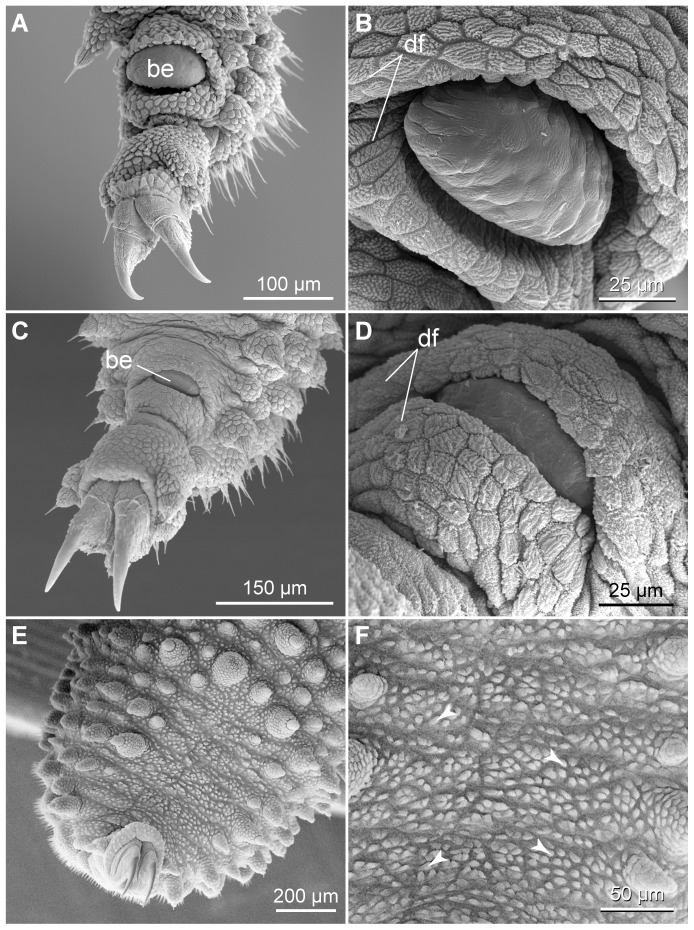
Scanning electron micrographs of structures associated with distal leg portions. Anterior is towards the lower right corner in all images. (A–D) Position and details of bean-shaped papillae. (A, B) *Principapillatus hitoyensis* **gen. et sp. nov.** (C, D) *Epiperipatus biolleyi*. Note that each bean-shaped papilla lies in a pouch formed by two folds. (E, F) Corresponding dorsal area of the leg in *Eoperipatus* sp. showing no trace of a bean-shaped papilla (image E). Note also the lack of dermal papillae in this area, which is instead covered with small granular scales (arrowheads in F). Abbreviations: be, bean-shaped papilla; df, dermal folds.

The structure of legs and feet in the two neotropical species also differs from that in *Eoperipatus* sp.. First, the proportion of leg to foot width (5∶1 to 6∶1) is clearly larger in *Eoperipatus* sp. as compared to the neotropical species (2∶1 to 3∶1 in both species). Second, *Eoperipatus* sp. shows only one anterior and one posterior distal foot papilla, whereas the neotropical species possess two anterior and one posterior papillae (Figure 18A–C). Third, a large, bean-shaped papilla is found on the dorsal leg surface above each foot in the neotropical species (Figure 19A–D). This papilla lies in a pouch formed by two conspicuous tegumental folds, which appear nearly closed in some specimens (Figure 19B, D). The bean-shaped papilla and its pouch are missing in *Eoperipatus* sp. and the corresponding region of the leg surface lacks dermal papillae but is covered instead with granular scales (Figure 19E, F).

The males of the three species show one pair of crural gland openings per leg in the two pregenital segments. However, the structure of the area surrounding each opening differs between *Eoperipatus* sp. and the two neotropical species. In *Epiperipatus biolleyi* and *Principapillatus hitoyensis* **gen. et sp. nov.**, there are two separate tubercles covered with scales and only their apices have a smooth surface (Figure 16A, B). In contrast, the paired crural tubercles are linked by a prominent dermal fold in *Eoperipatus* sp., thus forming a single complex covered with scales some of which are modified (Figure 16C: inset). These modified scales form a distinct anterior field on the surface of the crural complex (Figure 16C).

The number, position and structure of the anal gland openings differ in males from the two geographic regions. In the two neotropical species, the anal glands open to the exterior via a pair of slits, whereas the South-East Asian species of *Eoperipatus* shows a single large cruciform opening located on a specialised pad nearly as large as the genital pad (Figure 16D–F). In contrast to the neotropical species, the male genital pad of *Eoperipatus* sp. has four peculiar circular pits (Figure 15C). The female genital opening is also different in this species (Figure 15D), as it appears as a transverse rather than a longitudinal slit characteristic of the two neotropical species.

Among the embryonic structures, we found numerous projections covering the distal foot portions in embryos at advanced developmental stages in the neotropical species (Figure 18D, E). These foot projections are clearly missing in embryos of *Eoperipatus* sp., which instead show a smooth cuticle covering presumptive claws (Figure 18F).

### Characteristic features of the new Costa Rican genus and species

Since the new species shows a unique combination of characters, we assign it to a new genus and provide a formal description and designate types for both, the new genus and species to fulfil the requirements of the International Code of Zoological Nomenclature (ICZN).

*Principapillatus* **gen. nov.**

urn:lsid:zoobank.org:act:B745FE07-9DFE-4377-AD4B-F00D4CE2A8BE

Type species: *Principapillatus hitoyensis* **gen. et sp. nov.**, by monotypy.

Genus etymology: The name *Principapillatus* is derived from Latin *principalis* ( = main, primary) and *papillatus* ( = papilla-like, bud-like) and refers to the peculiar arrangement of dorsal primary papillae.

**Figure 20:**
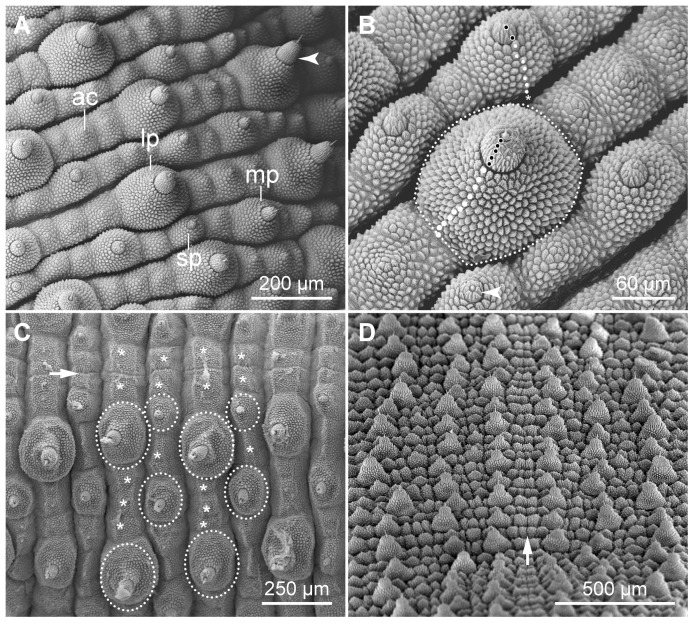
Scanning electron micrographs of dorsal integument in *Principapillatus hitoyensis* gen. et sp. nov. (A) Dorsolateral integument showing variation in size of primary papillae. Arrowhead points a slender primary papilla with a cylindrical apical piece on the lateral body surface. Median is left, posterior is towards the upper left corner. (B) Number of scale ranks and structure of primary papillae. Note the peculiar shape of the base in large primary papillae (dotted line). Note also the variable number of scale ranks in basal (white dots) and apical pieces (white dots with a black centre) of papillae of different size. Asterisk indicates the basal-most scale rank situated deep in the fold. Arrowhead points to a small primary papilla with a single scale rank in the apical piece. Anterior is towards the lower right corner, median is towards the lower left corner. (C) Distribution pattern of primary (white dotted line) and accessory papillae (asterisks) along the dorsomedian furrow (arrow). Note the repeated arrangement of dermal papillae along the dorsal midline and the constant number of only one accessory papilla on each side of the dorsomedian furrow. Anterior is left. (D) Overview of the longitudinal rows of large primary papillae along dorsal midline (arrow). Anterior is down. Abbreviations: ac, accessory papilla; lp, large primary papilla; mp, medium-sized primary papilla; sp, small primary papilla.

**Figure 21:**
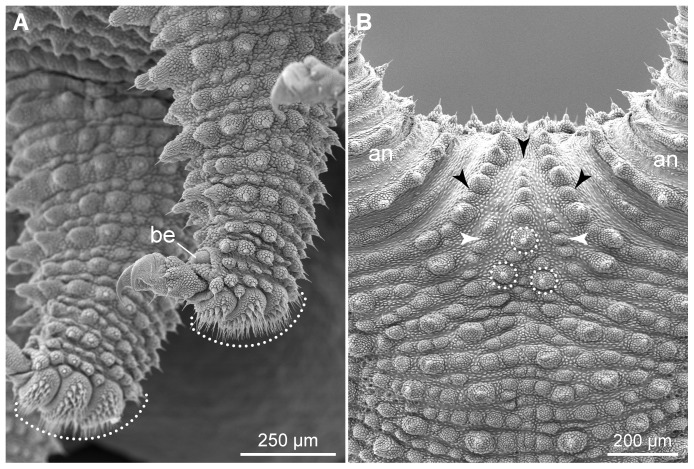
Scanning electron micrographs of additional features characteristic of *Principapillatus hitoyensis* gen. et sp. nov. (A) Position and size of the spinous pads. Note the small area occupied by the spinous pads, which is less than one third of the entire leg length. Dorsal is up, lateral is left. (B) Arrangement of dermal papillae on the head. Note the characteristic pattern of three large primary papillae (dotted lines) forming a triangle and additional rows of dermal papillae (black and white arrowheads). Anterior is up. Abbreviations: an, antenna; be, bean-shaped papilla.

Genus diagnosis: Primary papillae varying in size, with bases roundish anteriorly and posteriorly but straight medially and laterally (Figure 20B); largest and medium-sized primary papillae arranged in an alternated pattern: plica with largest primary and accessory papillae is followed by plica with medium-sized and smallest primary papillae and accessory papillae (Figures 4B, 20A–C); largest primary papillae organised in prominent rows parallel to dorsal midline (Figures 4B, 20D). Four straight spinous pads: first pad smaller than second and third pads, which are similar in size (Figures 5: inset, 13A); fourth pad smallest and missing in the anterior-most and two posterior-most leg pairs; fifth pad missing completely; area occupied by the spinous pads shorter than one third of entire leg length (Figures 5, 21A). One pair of separate, hemispherical interpedal structures per segment, covered with a finely granulated cuticle and surrounded by a collar of accessory papillae (Figures 5, 17A, B).

*Principapillatus hitoyensis* **gen. et sp. nov.**

urn:lsid:zoobank.org:act:F18DD6FC-A48E-403D-AC09-F5608720155A

(Figures 1A, B, 2A, 3–5, 6A, D, 7B, D, 8A, D, 9A, D–F, 10A, D, 11A, D, 12A, B, 13A, D, 14A, B, 15A, B, 16A, D, 17A, B, 18A, D, 19A, B, 20, 21, 22, 23; Data S1: Figures S1A, S2, S3, S4)

**Figure 22:**
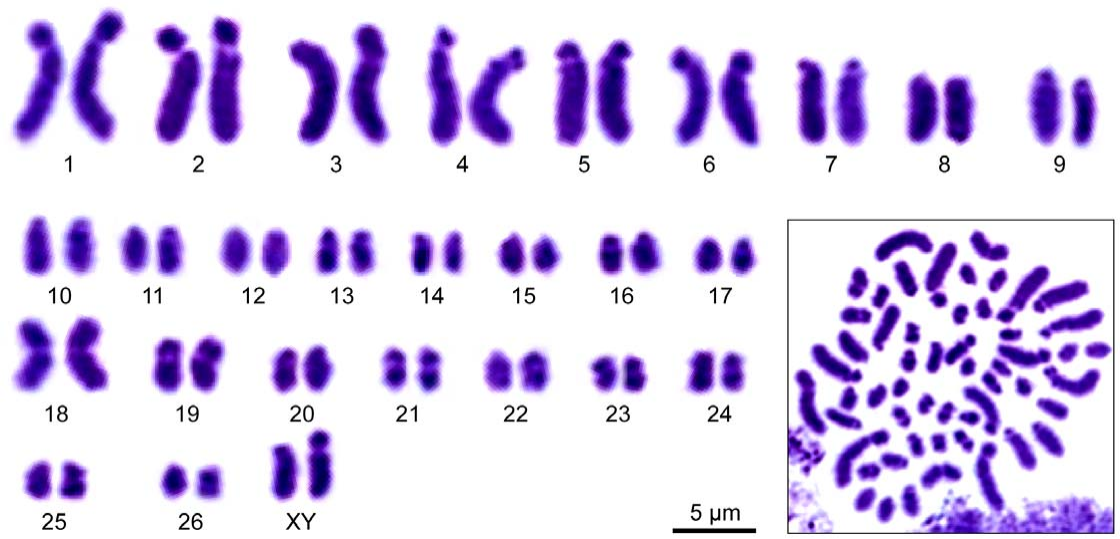
Karyotype of *Principapillatus hitoyensis* gen. et sp. nov. Inset in the lower right corner shows a light micrograph of the original preparation of mitotic chromosomes from a testis stained with Giemsa. Note the presence of a heteromorphic pair of sex chromosomes (XY). The pairs 1–17 and XY represent acrocentric chromosomes, whereas the pairs 18–26 comprise metacentric/submetacentric chromosomes.

**Figure 23:**
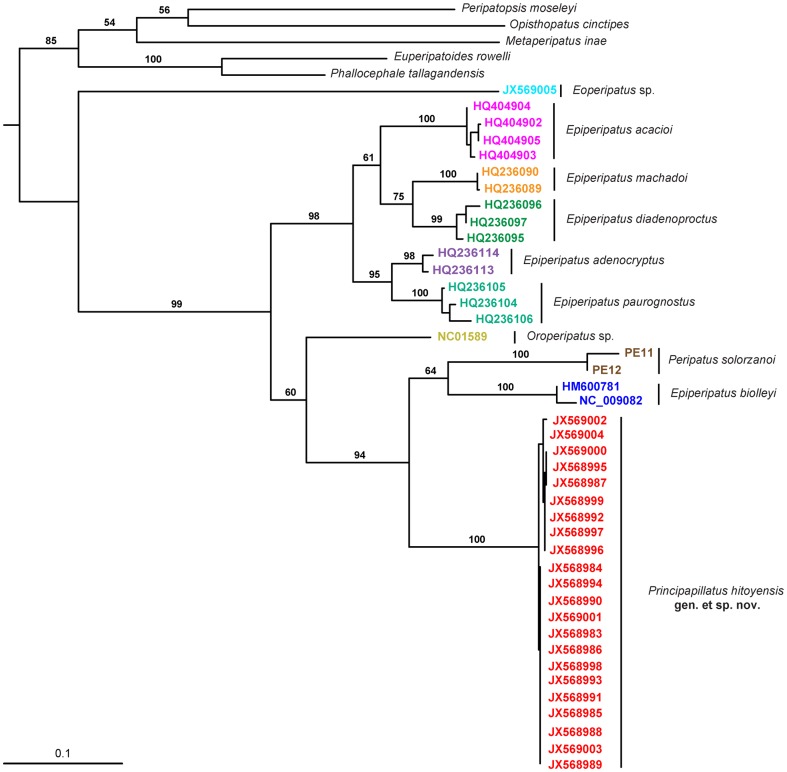
Phylogenetic relationships of different species of Peripatidae. Maximum Likelihood topology based on a combined analysis of *COI* and *12S rRNA* nucleotide sequences, with five peripatopsid species as an outgroup. Only the bootstrap values higher than 50 are shown. Abbreviations in different colours correspond to the accession numbers of the *COI* sequences in GenBank.

Previously referred to as “*Epiperipatus biolleyi*” [42]; “*Epiperipatus isthmicola* (Bouvier, 1902)” [43]–[48], and “*Epiperipatus* cf. *isthmicola*” [21], [49], [50].

Material examined: Holotype — male, in 70% ethanol; COSTA RICA, Limón, Reserva Biológica Hitoy Cerere, tropical rain forest, 09°40′21.56″N, 83°02′36.97″W, 300 m, 08–10 October 2005, G. Mayer col. (MZUCR63-01). Paratypes — a total of five males and five females obtained from culture established in 2005–2012 based on original specimens collected from type locality; two males and two females deposited in the MZUCR (MZUCR64–67-01); one male and one female deposited in each of the following collections: BMNH (BMNH(E)1038600, 1038601), UFMG (UFMG189, 190) and SNSD (S1, S2; *Evertebrata varia* section). Additional material — six adult females, two adult males, three juvenile females, one juvenile male and one embryo female were obtained from cultures and prepared for scanning electron microscopy. A total of 548 females and 561 males of different ages, all obtained from cultures, were used for leg counting as well as for determining the sex ratio and analysing the lifespan, colour variation and reproduction.

Species etymology: The species is named in reference to its type locality within the Reserva Biológica Hitoy Cerere. The name of the reserve includes the Indian words (Bribri language) *Hitoy*, meaning “green blanket” (referring to a dense forest covering the landscape), and *Cerere*, meaning “clear water” (referring to numerous rivers in the region).

Species diagnosis: Characteristic head pattern, with three large primary papillae forming a triangle and additional conspicuous rows of primary and accessory papillae (Figures 4B, 21B). Dorsomedian furrow flanked by one accessory papilla on each side, followed by one primary papilla (Figures 4B, 20C); large and medium-sized primary papillae arranged out of register along dorsal midline (Figures 4B, 20A, C). Embryonic foot projections barbed (Figure 18D). Males with 26–29, females with 30–32 leg pairs (Table 3). *COI* and *12S rRNA* sequences as in the 22 specimens sequenced (Table 2). Karyotype (2n = 54, XY) with 17 pairs of acrocentric and nine pairs of metacentric/submetacentric chromosomes, and an acrocentric heteromorphic sexual pair (Figure 22).

**Table 3:** Number of leg pairs in specimens of each sex in *Principapillatus hitoyensis* **gen. et sp. nov.**

**Number of leg pairs**	**26**	**27**	**28**	**29**	**30**	**31**	**32**
Number of males*	7	203	327	24	–	–	–
Number of females**	–	–	–	–	210	313	25

* Total number of analysed males: 561.

** Total number of analysed females: 548.

Description: The following description complements the data presented in previous sections of this paper. Ground colour of dorsal integument varying *in vivo* from dark-brown to reddish-orange, either with or without additional rhomboid pattern along dorsal midline; antennae and head region darker than the rest of the body (Figure 1A, B). Bright primary papillae concentrated laterally, forming longitudinal bands on each body side and short transverse stripe above each leg (Figure 1B). Legs greyish dorsally, with a single large primary papilla brighter than other papillae in the middle of third proximal transverse ring (Figure 1B). Ventral body surface pinkish-beige, with dark midline interrupted by bright spots corresponding to the preventral and ventral organs; dark spot present at the basis of each leg, proximal to coxal furrows. Preserved specimens brown, with the same pattern as in living specimens. Juveniles with the same colour and pattern variation as in adults.

Maximum body size after fixation in 70% ethanol: length up to 68.1 mm, width up to 6.5 mm, height up to 4.7 mm. Antennae with 41 to 46 complete antennal rings, 27 to 32 of which in antennal body and remaining 14 in antennal tip (including terminal button). Antennal sensory fields with spindle-shaped sensilla extend over 14–17 complete rings, beginning in the third-last ring of the proximal antennal region; however, additional 9–13 semi-rings with spindle-shaped sensilla are inserted between the complete rings; spindle-shaped sensilla with two ranks of long, flat scales and a blunt terminal bristle (Figure 3B). A pair of well-developed and pigmented eyes present posterolaterally to the antennae. Mouth surrounded by two rows of lips; internal row of six to seven pairs of lips and an unpaired anterior lip; external row with eight to ten pairs of small lips (Figures 3C, 9A). Outer and inner jaw blades with one principal and one accessory tooth; inner jaw blade with 9–12 denticles; accessory teeth of inner and outer jaw blades similar in shape to principal teeth (Data S1: Figure S2A).

First and last leg pairs reduced in size; last leg pair not rotated posteriorly and used for walking; first and penultimate leg pairs with three spinous pads, last leg pair with only two spinous pads. Eight transverse rings on each leg, alternated with thinner semi-rings between them; primary papillae larger on dorsal leg surface (Data S1: Figure S2B). Ventral foot surface with one bristle on the proximal and 1–2 bristles on the distal setiform ridges (Figure 18A). Embryos of advanced developmental stages with barbed foot projections (Figure 18D). Number of leg pairs not overlapping between sexes (Table 3).

Primary papillae with thorn-shaped, centred sensory bristles, which vary in size and bear a textured basis. Apical pieces of dorsal primary papillae cylindrical, but conical apical pieces present in lateral body regions (Figure 20A); apical pieces with 3–5 scale ranks in largest, 2–3 in medium-sized, and 1–2 in smallest primary papillae (Figure 20B). Basal pieces with 10–14 scale ranks in largest, 8–10 in medium-sized, and 5–9 in smallest primary papillae (Figure 20B). Type I crater-shaped papillae with 6–7 scales in the apical collar, type II papillae with 11–14 scales in the apical collar (Figure 11A, B).

Remarks on habitat and distribution: The species is only known from its type locality, the Reserva Biológica Hitoy Cerere [Hitoy-Cerere Biological Reserve], province of Limón, Costa Rica, which is a tropical rain forest situated ca. 200 km south-east from San José (09°40′21.56″N, 83°02′36.97″W), at an altitude of 300 m (Figure 2A). The adults were collected mostly in leaf litter, but some specimens were also found within rotten logs and among the roots of banana trees. One specimen was collected from an ant nest.

Remarks on reproduction: The reproductive data were obtained from specimens kept in culture. From time to time, we observed the posterior ends of males and females being connected and their genital openings pressed against each other, while the male held the female's posterior end with the last leg pair. We interpret this behaviour as mating, which suggests a direct transfer of sperm into the female genital opening. Females start giving births when they are 10–15 months old. No apparent seasonality is evident, as females give birth throughout the year (Data S1: Figure S3). Similar observations were made for *Epiperipatus biolleyi* and *Epiperipatus isthmicola* (Bouvier, 1902) from Costa Rica [51], whereas *Epiperipatus acacioi* from Brazil clearly shows seasonality coinciding with the wet season from December to July [52]. On average, each cultured female gave birth to 20–40 young during the entire lifespan, which can exceed five years. The number of males and females born in our cultures was equal, whereas the ratio of collected males to females was 1 to 4, as reported for other species of Peripatidae [53] and Peripatopsidae [54]–[56].

Remarks on phylogenetic relationships: Our alignment of the *COI* fragments contained 600 bp. The translation of the *COI* nucleotide sequences into amino acid sequences revealed no stop codons, suggesting that the sequences belong to functional mitochondrial protein-coding genes. The alignment of the *12S rRNA* fragments contained 478 bp. According to our results, the *COI* and *12S rRNA* sequences are A + T biased. The Maximum Likelihood analyses using either nucleotides or translated amino acids of *COI* revealed congruent topologies (Figure 23; Data S1: Figure S4). *Principapillatus hitoyensis* **gen. et sp. nov.** is retrieved as a monophyletic clade, showing a high bootstrap support value (100) and forming the sister group to a clade consisting of *Epiperipatus biolleyi* and *Peripatus solorzanoi* Morera-Brenes & Monge-Nájera, 2010 (Figure 23).

## Discussion

### Monophyly versus non-monophyly of the peripatid genera

This study describes a new onychophoran species from Costa Rica, *Principapillatus hitoyensis* **gen. et sp. nov.**, which shows a unique combination of characters not consistent with description for any of the ten existing genera of the Peripatidae [57] (Data S1: Table S2). In contrast to representatives of *Eoperipatus*, *Typhloperipatus*, *Oroperipatus* and *Heteroperipatus*, the new species shows one posterior and two anterior distal foot papillae (see refs [58]–[62]). Likewise, it cannot be assigned to *Mesoperipatus*, as it shows four rather than three spinous pads, to *Plicatoperipatus*, as it has only 12 rather than 24 dorsal plicae per segment, to *Speleoperipatus*, as it has a short and straight rather than a long and crescent-shaped fourth spinous pad, or to *Macroperipatus*, as it does not have the modified quadrangular dermal papillae characteristic of this genus [1], [2], [9], [22], [58], [59], [63], [64]. Moreover, the males of the new species bear crural tubercles only in two pregenital leg pairs, which contrasts with their putative occurrence in several leg-bearing segments in *Peripatus* [1], [9]. In addition, the variation in size and the characteristic shape of large and medium-sized dermal papillae differ from representatives of both *Peripatus* and *Epiperipatus* [2], [64].

The establishment of the new genus is supported by the results of our phylogenetic analyses according to which *Principapillatus hitoyensis* **gen. et sp. nov.** forms a sister group of *Epiperipatus biolleyi* and *Peripatus solorzanoi*. In contrast, *Epiperipatus* is revealed as a polyphyletic assemblage of species that variously group with representatives of *Oroperipatus*, *Peripatus* and *Principapillatus* **gen. nov.**. This finding is in line with morphological observations, which have provided no evidence for the monophyly of *Epiperipatus* [17], [57] (Data S1: Table S2). A thorough revision of *Epiperipatus* would be appropriate [57].

The problem of subjective classification is not restricted to *Epiperipatus*. Our survey through the literature revealed that among the ten genera of Peripatidae described previously, nine might show at least one unique or potentially derived character, although many of these characters have not been included in the original diagnoses of these genera (Data S1: Table S2). We used these characters to provide emended diagnoses for these nine genera of Peripatidae (Table 4). For example, *Eoperipatus* can be distinguished by the structure of crural, genital and anal gland openings in males, *Typhloperipatus* by details of genital tract and embryogenesis, and by rudimentary eyes, *Mesoperipatus* by separate ovarian tubes, three spinous pads per leg and contiguous anal gland openings, *Plicatoperipatus* by twenty-four dermal plicae per segment, and *Speleoperipatus* by complete lack of eyes and body pigmentation [1], [9], [58], [59], [61] (Table 4).

**Table 4:** Emended diagnoses of the peripatid genera based on putatively derived features.

Genus name	Characteristic features	References
*Eoperipatus* Evans	Males with a single and medial anal gland opening situated on a conspicuous pad located anterior to the anus; genital pad of males with four circular pits; crural tubercles linked by a dermal fold, forming a single complex on each leg of the two pregenital leg pairs (these crural complexes have been misinterpreted as coxal vesicles by Evans [59] and Sedgwick [8]); each crural complex is covered with two different types of scales (an anterior field of modified scales is clearly seen in scanning electron micrographs)	Evans [59]; present study
*Epiperipatus* (Clark)	*	–
*Heteroperipatus* Zilch	One posterior and three anterior distal foot papillae on each foot	Zilch [62]
*Macroperipatus* (Clark)	Dermal papillae of dorsal integument flattened, with quadrangular bases, and covered with flat scales that are embedded into the papilla surface; primary papillae with vestigial apical pieces showing a single-ranked collar of reduced scales	Read [2], [64]; Oliveira *et al.* [22]
*Mesoperipatus* Evans	Male anal gland openings close together in a single medial groove anterior to the anus, but separated by a tegumental fold; three spinous pads per leg; ovarian tubes completely separate	Evans [59]; Bouvier [9]; Mayer & Tait [43]
*Oroperipatus* (Cockerell)	Two or more anterior and two or more posterior distal foot papillae (altogether 4–7 distal foot papillae)	Bouvier [9]
*Peripatus* Guilding	Crural tubercles present in more than two pregenital leg pairs in males; apical piece of dorsal primary papillae larger than the basal piece	Bouvier [9]; Peck [1]; Read [2]
*Plicatoperipatus* (Clark)	Twenty-four dorsal plicae per segment, which show numerous anastomoses; apical-most scales of basal piece thorn-shaped, as high as the apical piece and sticking out	Bouvier [9]; Clark [58]; Read [2]
*Speleoperipatus* Peck	Eyes not visible externally (internal head structure not analysed); body pigmentation lacking completely	Peck [1]
*Typhloperipatus* Kemp	Eyes not visible externally, but rudimentary optic vesicles are present internally; oviducts and ovarian lumen fused for a long distance in front of the ovary; uterine embryos of nearly the same age	Kemp [61]; Mayer & Tait [43]

Note that no characteristic features can be provided for *Epiperipatus* (asterisks). See also Data S1: Table S2.

* No unique features were identified in representatives of *Epiperipatus* (see Data S1: Table S2).

Representatives of *Macroperipatus* are characterised by highly modified, flattened and quadrangular dermal papillae with reduced and flat scales and this feature might be present only in the type species of the genus, *Macroperipatus torquatus* (von Kennel, 1883) [2], [22], [64]. If future studies confirm that the remaining species assigned to *Macroperipatus* do not have this type of papillae, they will have to be excluded from this genus to retain its monophyly [22]. The distribution of crural tubercles in more than two (up to nine) pregenital leg pairs and the presence of dorsal primary papillae with large apical pieces (larger than basal pieces) might be the unique features of *Peripatus* [1], [9]. However, again, these features were reported only from a few species assigned to this genus [9]. Likewise, the number of three anterior and one posterior distal foot papillae might be a characteristic feature of *Heteroperipatus* [62], provided that *Heteroperipatus clarki* (Dunn, 1943) is excluded from this genus [57]. *Oroperipatus* can be recognised by the existence of at least two anterior and two posterior distal foot papillae, although the number of these structures varies from four to seven among the species assigned to this genus. The remaining peripatid genus, *Epiperipatus*, shows either ambiguous or overlapping characters with other genera (Data S1: Table S2). Hence, analysing additional features in all species assigned to this genus would help clarify the monophyly of *Epiperipatus sensu stricto* and detect additional monophyletic clades within this artificial assemblage.

### The utility of unexplored characters for taxonomic and phylogenetic studies of Onychophora

Our study revealed various structures on the onychophoran body surface the function of most of which is unknown. While different types of antennal sensilla, crater-shaped papillae, bean-shaped papillae and frontal organs might be sensory structures, the functions (if any) of interpedal structures, ventral and preventral organs, coxal vesicles, the postoral pit and fields of modified scales on the ventral body surface are unknown. A putative involvement of the embryonic foot projections in the uptake of material from the lumen of the uterus was suggested by Walker & Campiglia [11], [12], but this function requires corroboration.

Despite the uncertain function of the embryonic foot projections, structural differences between the species might be useful for taxonomic studies of the neotropical Peripatidae. Embryonic foot projections have been reported from *Epiperipatus acacioi* (Marcus & Marcus, 1955), *Epiperipatus biolleyi*, *Epiperipatus edwardsii* (Blanchard, 1847), *Macroperipatus torquatus*, *Oroperipatus corradoi* (Camerano, 1898) and *Principapillatus hitoyensis* **gen. et sp. nov.** (present study; refs [9]–[12]). Notably, the embryos of *Principapillatus hitoyensis* **gen. et sp. nov.** and *Epiperipatus acacioi* have barbed projections, whereas these are smooth in *Epiperipatus biolleyi* (present study; refs [11], [12]). The condition in the remaining species is unknown, as these structures have not been analysed using scanning electron microscopy [9], [10]. Clarifying this aspect in additional species might reveal a putative monophyletic clade within the neotropical Peripatidae. The results of our phylogenetic analyses indicate that the smooth projections are a derived feature of a clade that includes *Epiperipatus biolleyi*, whereas the barbed projections might represent an ancestral state, as they occur in two distantly related species, *Principapillatus hitoyensis* **gen. et sp. nov.** and *Epiperipatus acacioi*.

Our data further revealed that additional features, such as the bases of bristles on the spinous pads, the interpedal structures and the arrangement of head papillae, differ among species and, therefore, might be informative at species or genus level. Thus, more attention should be paid to these features in studies of additional species.

So far, karyotypic data have not been used widely for taxonomic studies of the peripatid species. However, chromosomal data have proven useful among the species of Peripatopsidae [13]–[15]. In these studies, enormous diversity in chromosome number and size distribution was revealed, and chromosomal information has been incorporated into species descriptions [13]. Indeed, in some cases, chromosomal differences provided the initial evidence for the existence of cryptic species [14], which were subsequently characterised on morphological grounds [3]. Unfortunately, peripatopsid chromosomes have proved intractable to traditional banding techniques, and without useful markers to establish synteny, it is not possible to identify the nature of the rearrangements involved in their evolution (see [15] for an exception). However, if modern hybridisation techniques prove useful in revealing precise patterns of chromosomal change, karyotypic data may similarly be useful not only for establishing species status, but also for reconstructing phylogeny.

The results reported here show that peripatids possess higher and lower chromosome numbers than any of the approximately 40 peripatopsid species reported to date, suggesting that enormous karyotypic diversity occurs in this onychophoran subgroup. Our data show that *Principapillatus hitoyensis* **gen. et sp. nov.** (2n = 54, XY) clearly differs from *Epiperipatus biolleyi* (2n = 64) both in chromosome number, and the presence of recognisable sex chromosomes. These two species are clearly distinct from *Eoperipatus* sp., which shows a small number of 2n = 8 large chromosomes (Data S1: Figure S5). Hence, karyological data promise to be a useful tool in future taxonomic characterisation of the Peripatidae.

In summary, our findings demonstrate that there is a diversity of characters that has not been explored sufficiently in Onychophora. Analysing characters described herein in additional species, in particular of the Peripatidae, might help clarify the diversity and phylogeny of this putatively uniform, albeit speciose, onychophoran taxon [17]. This approach might also reveal additional characters and character states that are absent in the three species examined herein and which could be used to recognise additional monophyletic clades. Some of these characters might also prove useful for studying the evolutionary relationships of the Peripatopsidae, the second major onychophoran subgroup.

## Supporting Information

Data S1 **Supplementary file containing the following figures and tables.**: Figure S1. Eversible coxal vesicles (arrowheads). (A) *Principapillatus hitoyensis* **gen. et. sp. nov.** Light micrograph of ventral leg surface. (B) *Eoperipatus* sp.. Scanning electron micrograph of ventral leg surface. Anterior is right in both images. Figure S2. Additional features of *Principapillatus hitoyensis* **gen. et sp. nov.** (A) Characteristics of the inner and outer jaw blades. (B) Arrangement of transverse rings on legs. Anterior is left. Note the presence of thin semi-rings (arrowheads) between the complete rings (white dots). Circular inset shows an enlarged primary papilla. Abbreviations: at, accessory tooth; be, bean-shaped papillae; dt, denticles; ib, inner jaw blade; ob, outer jaw blade; pt, principal tooth. Figure S3. Number of births during the lifespan in four females of *Principapillatus hitoyensis* **gen. et sp. nov.** Lifespan is represented by horizontal lines; number of births is illustrated by vertical bars. The left and right filled circles associated with horizontal lines indicate birth and death of each female, respectively. Figure S4. Maximum Likelihood topology illustrating the phylogenetic relationships of several species of Peripatidae. Combined analysis of nucleotide sequences of *12S rRNA* and translated aminoacids of *COI*, with five peripatopsid species as an outgroup. Bootstrap values lower than 50 are not shown. Abbreviations correspond to the accession numbers of the *COI* sequence in GenBank. Figure S5. Karyotype of *Eoperipatus* sp. Inset shows a light micrograph of the original preparation of mitotic chromosomes from a testis stained with Giemsa. Note three pairs of large and one pair of small chromosomes. Table S1. Number of specimens analysed using each method. Numbers are provided for specimens of different sexes and ages, including embryos. Table S2. Current inconsistencies with the diagnostic features used for the peripatid genera. Symbols used are as follows: (+) present, (−) absent, (+/−) found only in some species of the genus, (?) data unavailable. Grey shading highlights potentially unique features for each genus (note that most proposed diagnostic features are not unique and have to be revised).
(PDF)
