# Supplementary material for: Unexplored Character Diversity in Onychophora (Velvet Worms): A Comparative Study of Three Peripatid Species
Source: PLoS One. 2012 Dec 17;7(12):e51220. doi: 10.1371/journal.pone.0051220 (PMC3524137; doi:10.1371/journal.pone.0051220)
Supplement: Data S1 — Supplementary file containing the following figures and tables.: Figure S1. Eversible coxal vesicles (arrowheads). (A) Principapillatus hitoyensis gen. et. sp. nov. Light micrograph of ventral leg surface. (B) Eoperipatus sp.. Scanning electron micrograph of ventral leg surface. Anterior is right in both images. Figure S2. Additional features of Principapillatus hitoyensis gen. et sp. nov. (A) Characteristics of the inner and outer jaw blades. (B) Arrangement of transverse rings on legs. Anterior is left. Note the presence of thin semi-rings (arrowheads) between the complete rings (white dots). Circular inset shows an enlarged primary papilla. Abbreviations: at, accessory tooth; be, bean-shaped papillae; dt, denticles; ib, inner jaw blade; ob, outer jaw blade; pt, principal tooth. Figure S3. Number of births during the lifespan in four females of Principapillatus hitoyensis gen. et sp. nov. Lifespan is represented by horizontal lines; number of births is illustrated by vertical bars. The left and right filled circles associated with horizontal lines indicate birth and death of each female, respectively. Figure S4. Maximum Likelihood topology illustrating the phylogenetic relationships of several species of Peripatidae. Combined analysis of nucleotide sequences of 12S rRNA and translated aminoacids of COI, with five peripatopsid species as an outgroup. Bootstrap values lower than 50 are not shown. Abbreviations correspond to the accession numbers of the COI sequence in GenBank. Figure S5. Karyotype of Eoperipatus sp. Inset shows a light micrograph of the original preparation of mitotic chromosomes from a testis stained with Giemsa. Note three pairs of large and one pair of small chromosomes. Table S1. Number of specimens analysed using each method. Numbers are provided for specimens of different sexes and ages, including embryos. Table S2. Current inconsistencies with the diagnostic features used for the peripatid genera. Symbols used are as follows: (+) present, (−) [file pone.0051220.s001.pdf]

Supporting Information

Unexplored Character Diversity in Onychophora (Velvet Worms): a Comparative Study of Three Peripatid Species

Ivo de Sena Oliveira, Franziska Anni Franke, Lars Hering, Stefan Schaffer, David M. Rowell, Andreas Weck-Heimann, Julián Monge-Nájera, Bernal Morera-Brenes & Georg Mayer

Supporting Figures

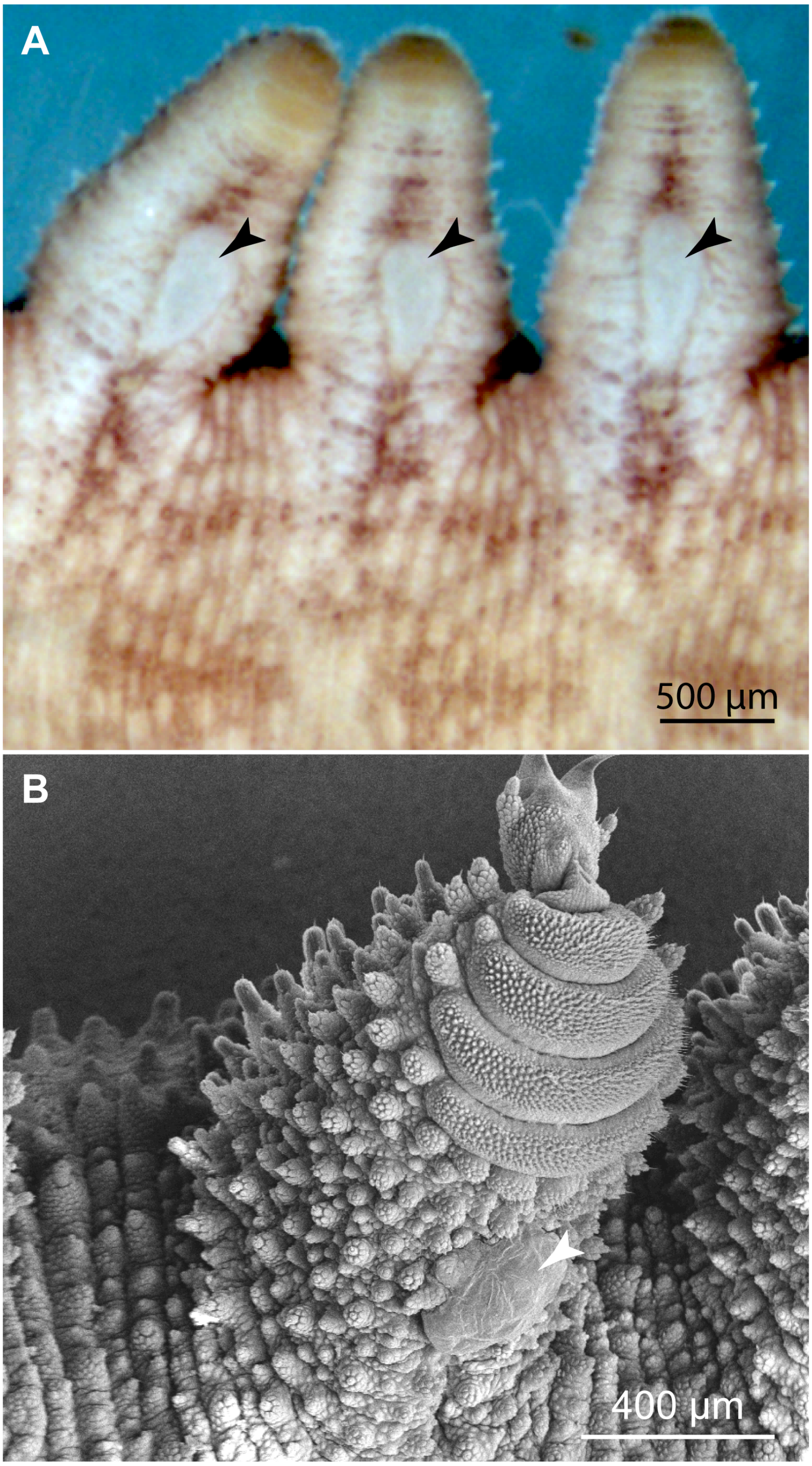

Figure S1. Eversible coxal vesicles (arrowheads). (A) *Principapillatus hitoyensis* **gen. et. sp. nov..** Light micrograph of ventral leg surface. (B) *Eoperipatus* sp.. Scanning electron micrograph of ventral leg surface. Anterior is right in both images.

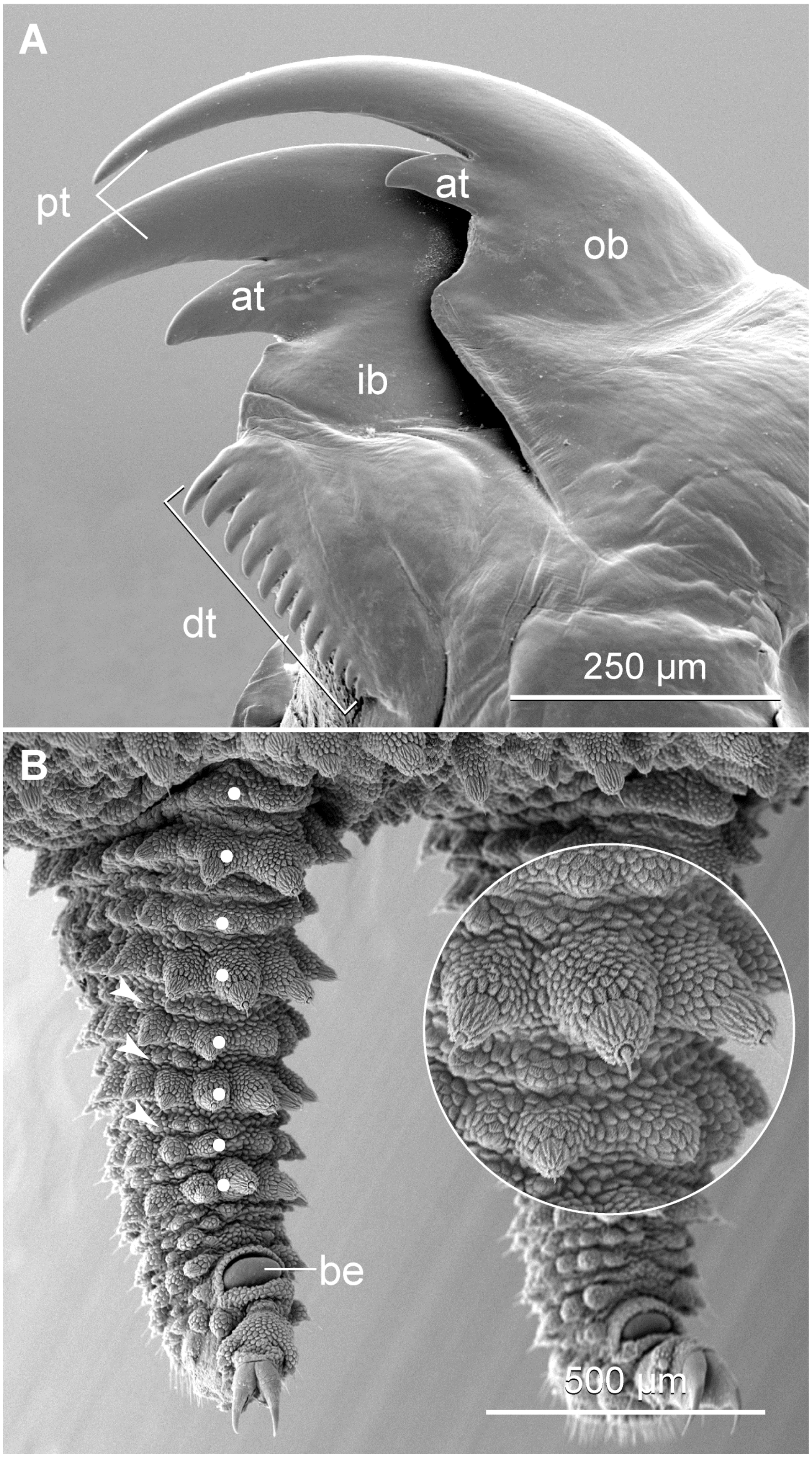

Figure S2. Additional features of *Principapillatus hitoyensis* gen. et sp. nov.. (A) Characteristics of the inner and outer jaw blades. (B) Arrangement of transverse rings on legs. Anterior is left. Note the presence of thin semi-rings (arrowheads) between the complete rings (white dots). Circular inset shows an enlarged primary papilla. Abbreviations: at, accessory tooth; be, bean-shaped papillae; dt, denticles; ib, inner jaw blade; ob, outer jaw blade; pt, principal tooth.

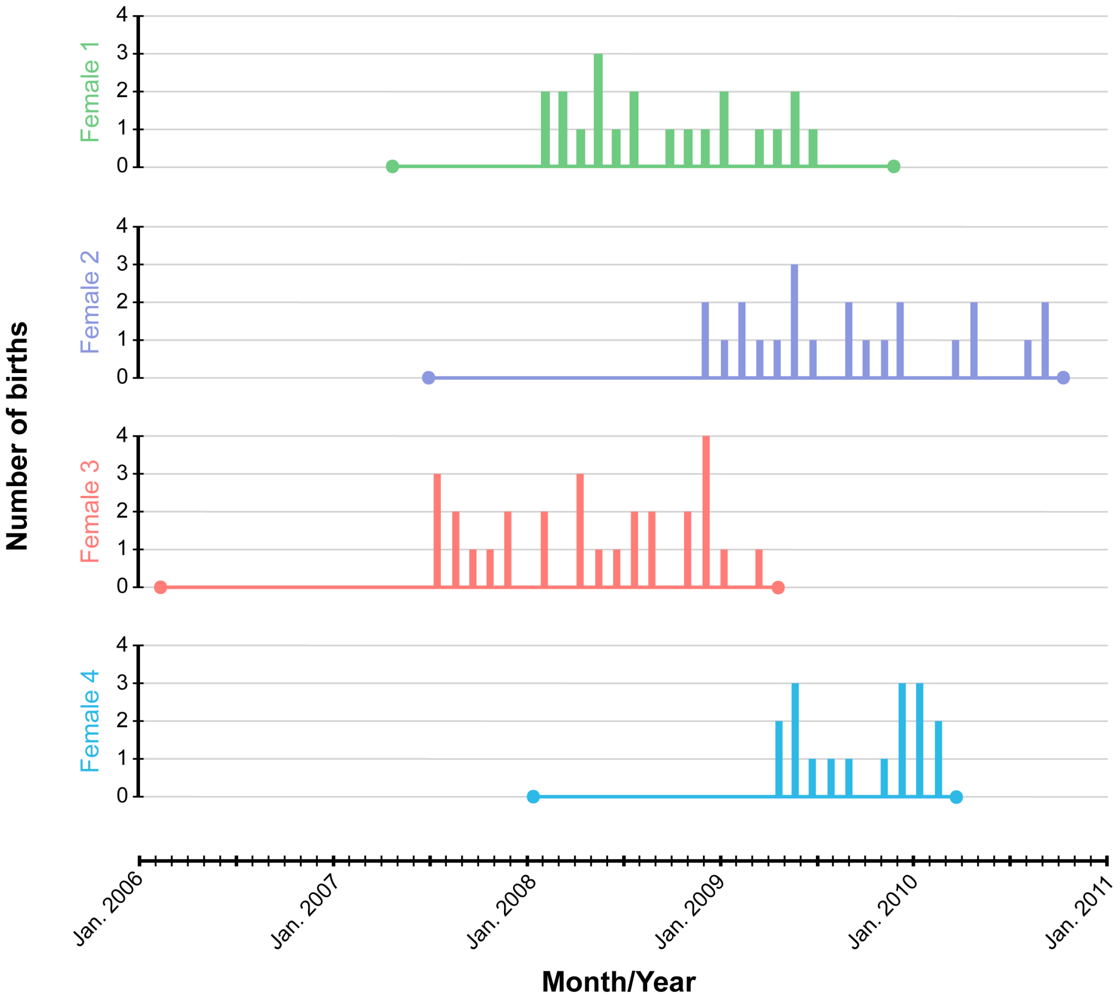

Figure S3. Number of births during the lifespan in four females of *Principapillatus hitoyensis* gen. et sp. nov.. Lifespan is represented by horizontal lines; number of births is illustrated by vertical bars. The left and right filled circles associated with horizontal lines indicate birth and death of each female, respectively.

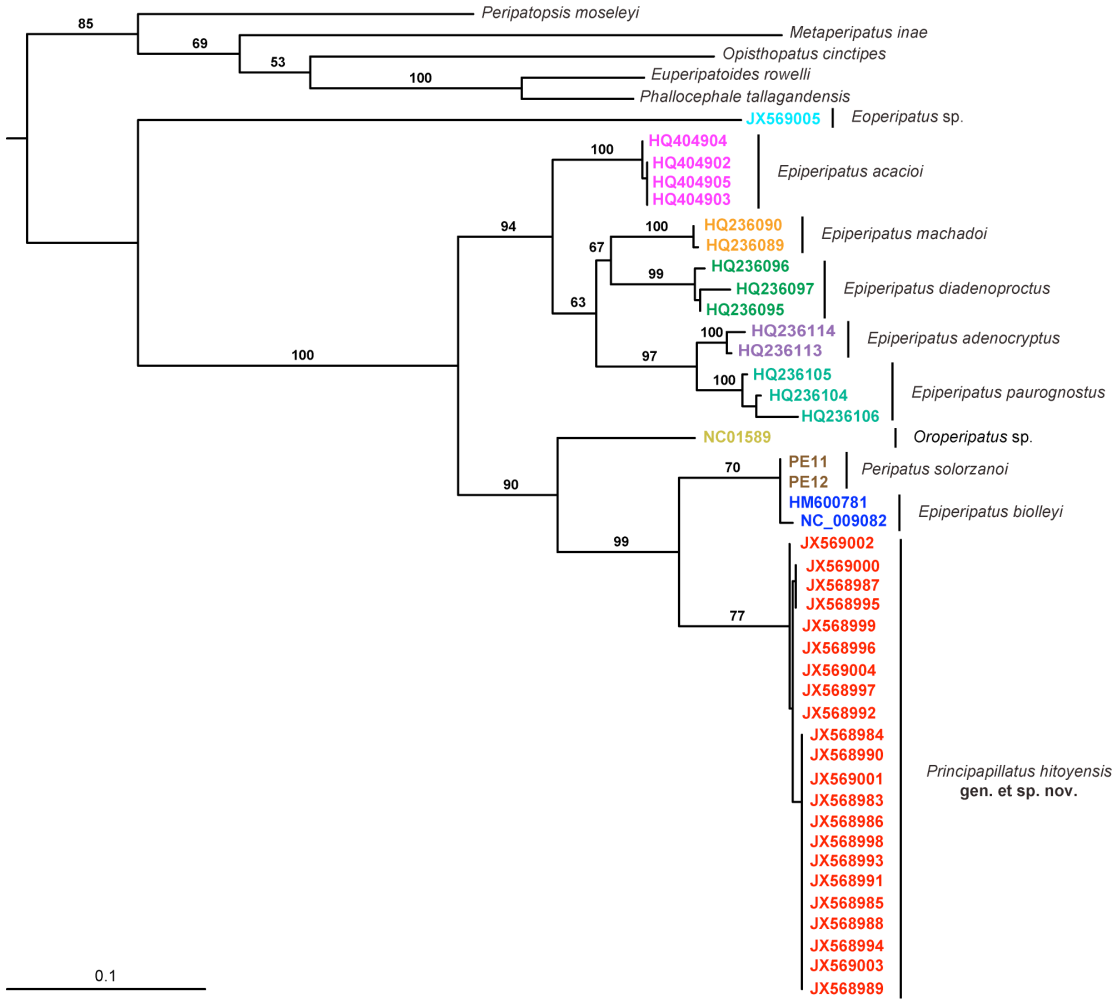

Figure S4. Maximum Likelihood topology illustrating the phylogenetic relationships of several species of Peripatidae. Combined analysis of nucleotide sequences of *12S rRNA* and translated aminoacids of *COI*, with five peripatopsid species as an outgroup. Bootstrap values lower than 50 are not shown. Abbreviations correspond to the accession numbers of the *COI* sequence in GenBank.

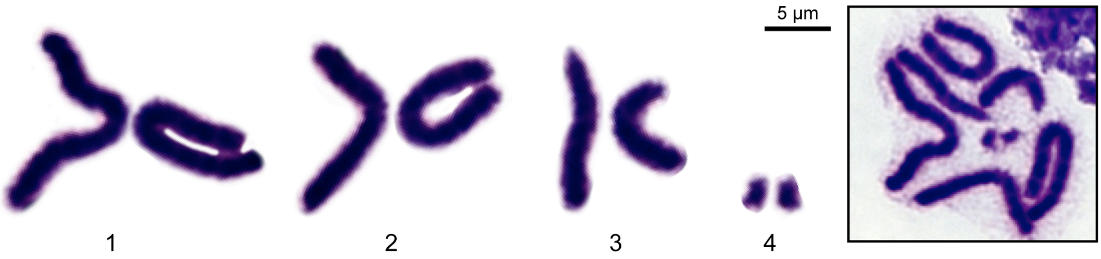

Figure S5. Karyotype of *Eoperipatus* sp.. Inset shows a light micrograph of the original preparation of mitotic chromosomes from a testis stained with Giemsa. Note three pairs of large and one pair of small chromosomes.

Supporting Tables

Table S1. Number of specimens analysed using each method. Numbers are provided for specimens of different sexes and ages, including embryos.

**Method**	**Number of specimens analysed**		
	Principapillatus hitoyensis **gen. et sp. nov.**	*Epiperipatus biolleyi*	*Eoperipatus* sp.
Analyses of colour pattern	1109	16	66
Counting of leg pairs	1109	16	66
Light microscopy	~20	~5	~10
Scanning electron microscopy	14	9	6
Karyology	5	–	3
Molecular analyses	21	–	1

Table S2. Current inconsistencies with the diagnostic features used for the peripatid genera. Symbols used are as follows: (+) present, (−) absent, (+/−) found only in some species of the genus, (?) data unavailable. Grey shading highlights potentially unique features for each genus (note that most proposed diagnostic features are not unique and have to be revised).

**Diagnostic features of each genus, as suggested in the original descriptions and subsequent revisions (see References below)**	**Occurrence of the diagnostic features among the genera, as found in the descriptions of species assigned to each genus (see References listed below)**									
***Eoperipatus* Evans, 1901**	*Eoperipatus*	*Epiperipatus*	*Heteroperipatus*	*Macroperipatus*	*Mesoperipatus*	*Oroperipatus*	*Peripatus*	*Plicatoperipatus*	*Speleoperipatus*	*Typhloperipatus*
Four or five spinous pads per leg	+	+	+	+	−	+	+	+	+	+
Nephridial tubercle in the middle of the 4th pad or on its proximal side	+/−	−	−	−	−	−	−	−	−	−
4th and 5th leg pairs with only four spinous pads	+	+	+	+	−	+/−	+	+	+	+
One anterior and one posterior distal foot papillae	+	−	−	−	−	−	−	−	−	+
One or more bristles on each setiform ridge	+	+	+	+	+	+	+	+	+	+
Inner jaw blade with a diastema	+	+	+	+	+	+	+	+	+	+
Two accessory teeth on outer jaw blade	+	+/−	+/−	+/−	+/−	+/−	+/−	+/−	−	+/−
Up to three accessory teeth on inner jaw blade	+	−	−	−	−	+/−	−	−	−	+
Male genital opening cross-shaped	+	+/−	?	+	+	+	+	+	?	+
Single transverse opening on female genital pad	+	−	−	−	+	−	−	−	−	−
Ductus ejaculatorius long, forming a loop	+	+	?	+	+	+	+	+	?	+
Ovarian funnels (="receptacula ovorum") and receptacula seminis present	+	+	?	+	+/−	+	+	+	?	+
Ovary large and spreads out over the organs	+	−	?	−	−	−	−	−	?	+
Ova large, full of yolk and exogenous	+	−	?	−	−	−	−	−	?	+
Embryos without placental structures	+	−	?	−	−	−	−	−	?	+
Single and medial anal gland opening situated between the last pair of legs	+	−	?	−	−	−	−	−	?	−

***Epiperipatus* (Clark, 1913)**	*Eoperipatus*	*Epiperipatus*	*Heteroperipatus*	*Macroperipatus*	*Mesoperipatus*	*Oroperipatus*	*Peripatus*	*Plicatoperipatus*	*Speleoperipatus*	*Typhloperipatus*
Twelve dorsal plicae per segment separated by continuous furrows	+	+	+	+	+	+	+	−	+	+
Incomplete or bifurcated dorsal plicae in the space between two legs	+	+/−	+	+	−	+/−	+	+	−	−
Dorsal primary papillae all of one type	−	+	?	+/−	+	−	−	−	−	?
Dorsal primary papillae with rounded bases	+	+	+	−	+	+	+	+	+	+
Dorsal primary papillae intergraded through all stages in medium-sized and large specimens, while certain ones are markedly prominent in small specimens	+/−	+	?	+/−	+	+/−	+	+	+	?
Dorsal primary papillae close together, although accessory papillae may occur between them	+	+	+	+	+	+/−	−	+	−	?
Accessory papillae very diversely developed	+	+	?	−	+	+/−	+/−	−	?	?
Crural tubercles present only in two pregenital leg pairs in males	+	+	?	+	+	+	+/−	+	?	+
Apical piece of dorsal primary papillae with 2−3 scale ranks	+/−	+/−	?	+/−	?	−	−	+/−	?	?
***Heteroperipatus* Zilch, 1954**	*Eoperipatus*	*Epiperipatus*	*Heteroperipatus*	*Macroperipatus*	*Mesoperipatus*	*Oroperipatus*	*Peripatus*	*Plicatoperipatus*	*Speleoperipatus*	*Typhloperipatus*
27−28 leg pairs in males	−	+/−	+	+/−	−	+/−	+/−	−	−	−
31−32 leg pairs in females	−	+	+	+	−	+	+	−	−	−
Three anterior and one posterior distal foot papillae	−	−	+	−	−	−	−	−	−	−
Two anterior and two posterior distal foot papillae	−	−	+[1]	−	−	+	−	−	−	−
Four or five spinous pads	+	+	+	+	−	+	+	+	+	+
Nephridial tubercle in the 4th and 5th leg pairs below the 3rd spinous pad	+/−	+	+	+	+	−	+	+	+	−
Nephridial tubercle "more or less" (Zilch 1954b) separated from the 3rd spinous pad	+/−	+	+	+	+	−	+	+	+	−
***Macroperipatus* (Clark 1913)**	*Eoperipatus*	*Epiperipatus*	*Heteroperipatus*	*Macroperipatus*	*Mesoperipatus*	*Oroperipatus*	*Peripatus*	*Plicatoperipatus*	*Speleoperipatus*	*Typhloperipatus*
Twelve dorsal plicae per segment separated by continuous furrows	+	+	+	+	+	+	+	−	+	+
Incomplete or bifurcated dorsal plicae in the space between two leg pairs	+	+/−	+	+	−	+/−	+	+	−	−
Dorsal primary papillae with quadrangular bases	−	−	−	+/−	−	−	−	−	−	−
Dorsal primary papillae separated by longitudinal grooves parallel to the main body axis	−	−	−	+	−	+/−	−	−	−	−
Accessory papillae "well reduced in size and few in number" (Peck 1975).	−	+/−	−	+	−	+/−	+/−	−	−	−
Crural tubercles present in two pregenital leg pairs in males (at least in *M. perrieri*)	+	+	?	+	+	+	+/−	+	?	+

***Mesoperipatus* Evans, 1901**	*Eoperipatus*	*Epiperipatus*	*Heteroperipatus*	*Macroperipatus*	*Mesoperipatus*	*Oroperipatus*	*Peripatus*	*Plicatoperipatus*	*Speleoperipatus*	*Typhloperipatus*
Three spinous pads per leg	–	–	–	–	+[2]	–	–	–	–	–
Nephridial tubercle indenting the third pad	+/–	+/–	–	+/–	+	–	+/–	+	+	–
22–25 leg pairs	+	+/–	–	–	+	+/–	–	–	+	–
Single accessory tooth on both jaw blades	+/–	+/–	+	+/–	+	+/–	+/–	+/–	+/–	–
Inner jaw blade with a long row of denticles	+	+	+	+	+	+	+	+	+	+
Dorsal plicae complete	–	+/–	–	–	+	–	–	–	+	+
A pair of receptacula seminis in females	+	+	?	+	+	+	+	+	?	+
Presumable lack of ovarian funnels	–	–	?	–	–[3]	–	–	–	?	–
Male anal gland openings in front of the anus in a common groove	–	–	?	–	+	–	–	–	?	–
Two anterior and one posterior distal foot papillae	–	+	–	+	+	–	+	+	+	–

***Oroperipatus* Cockerell, 1908**	*Eoperipatus*	*Epiperipatus*	*Heteroperipatus*	*Macroperipatus*	*Mesoperipatus*	*Oroperipatus*	*Peripatus*	*Plicatoperipatus*	*Speleoperipatus*	*Typhloperipatus*
Two distinct anal gland openings in front of the anus	–	+	?	+	–	+	+	+	?	+
4–7 distal foot papillae	–	–	+	–	–	+	–	–	–	–
At least four spinous pads	+	+	+	+	–	+	+	+	+	+
Nephridial tubercles inserted in the third pad	–	–	–	–	–	+	–	–	–	+

***Peripatus* Guilding, 1826**	*Eoperipatus*	*Epiperipatus*	*Heteroperipatus*	*Macroperipatus*	*Mesoperipatus*	*Oroperipatus*	*Peripatus*	*Plicatoperipatus*	*Speleoperipatus*	*Typhloperipatus*
Dorsal primary papillae varying in size in all ages	–	+	?	+/–	+	+/–	+	–	+	?
Primary papillae predominantly cylindrical in shape	–	–	?	–	–	+/–	+	–	?	?
Conical accessory papillae smaller than the primary papillae	–	–	?	–	–	+/–	+	–	?	?
Usually three accessory papillae between two primary papillae	+/–	+/–	+/–	–	–	+/–	+	–	–	?
Primary papillae separated by rather broad intervals with accessory papillae	–	–	–	–	–	+/–	+	–	?	+
Crural tubercles in males usually present in more than two pregenital leg pairs	–	–	–	–	–	–	+	–	–	–
Apical piece of primary papillae with four or more scale ranks	+/–	–	?	–	?	+	+	–	?	?

***Plicatoperipatus* (Clark, 1913)**	*Eoperipatus*	*Epiperipatus*	*Heteroperipatus*	*Macroperipatus*	*Mesoperipatus*	*Oroperipatus*	*Peripatus*	*Plicatoperipatus*	*Speleoperipatus*	*Typhloperipatus*
24 plicae per segment	–	–	–	–	–	–	–	+	–	–
Plicae very indistinct, with numerous and irregular anastomoses	–	–	–	–	–	–	–	+	–	–
Dorsal primary papillae small and “subequal” (Clark 1913)	–	+/–	?	+/–	–	–	–	+	–	–
Accessory papillae rare or absent	–	+/–	–	+/–	–	+/–	–	+	–	–
Crural tubercles present in two pregenital leg pairs in males	+	+	?	+	+	+	+/–	+	?	+

***Speleoperipatus* Peck, 1975**	*Eoperipatus*	*Epiperipatus*	*Heteroperipatus*	*Macroperipatus*	*Mesoperipatus*	*Oroperipatus*	*Peripatus*	*Plicatoperipatus*	*Speleoperipatus*	*Typhloperipatus*
22 or 23 leg pairs	+	–	–	–	+	+/–	–	–	+	–
One posterior and two anterior distal foot papillae	–	+	–	+	+	–	+	+	+	–
Nephridial tubercle broadly attached to and indenting the 3rd spinous pad	+/–	+/–	–	+/–	–	–	+/–	+	+	–
Fourth spinous pad crescent-shaped	+	+	+	+	–	+	+	+	+	+
Fifth spinous pad absent	–	+/–	–	+/–	+	–	+/–	+	+	–

***Typhloperipatus* Kemp, 1913**	*Eoperipatus*	*Epiperipatus*	*Heteroperipatus*	*Macroperipatus*	*Mesoperipatus*	*Oroperipatus*	*Peripatus*	*Plicatoperipatus*	*Speleoperipatus*	*Typhloperipatus*
19 or 20 leg pairs	−	−	−	−	−	−	−	−	−	+
Number of leg pairs variable	+	+	+	+	+	+	+	+	+	+
Inner jaw blade with a diastema and a row of denticles	+	+	+	+	+	+	+	+	+	+
Four spinous pads per leg	+	+	−	+	−	+/−	+	+	+	+
Nephridial tubercle on the third pad	−	−	−	−	−	+	−	−	−	+
One anterior and one posterior distal foot papillae	+	−	−	−	−	−	−	−	−	+
Genital pad between the penultimate leg pair	+	+	+	+	+	+	+	+	+	+
Receptacula seminis present, with two ducts opening into the oviducts	+	+	?	+	+	+	+	+	?	+
Ovarian funnels present	+	+	?	+	−	+	+	+	?	+
Oviducts united for a long distance in front of the ovary	−	−	?	−	−	−	−	−	?	+
Ovaries completely fused, with a single cavity	+	−	?	−	−	−	−	−	?	+
Ovaries closely pressed against but not directly attached to the floor of the pericardium to which they are connected posteriorly by means of a ligament (=“funiculus”)	+	+	+	+	−[4]	+/−[4]	+	+	?	+
Ovary exogenous	+	−	?	−	−	−	−	−	?	+
Ova large and heavily charged with yolk	+	−	?	−	−	−	−	−	?	+
Ova about 5 mm in their longest diameter	+	−	?	−	−	−	−	−	?	+
Embryo without placental structures (referred to as “trophic vesicle” in the original description)	+	−	?	−	?	−	−	−	?	+
Uterine embryos of about the same age	−	−	?	−	−	−	−	−	?	+[5]
Spermatophores long, with horny coat and cap	+	+	?	?	?	?	?	?	?	+
Skin pigment brown, soluble in alcohol	+	+/−	+/−	+/−	+	+/−	+/−	+	−	+
Welldeveloped coxal vesicles	+	+	?	+	+	+	+	+	?	+
Single crural gland per leg in the two pre-genital leg pairs in males	−	+/−	?	−	−	−	−	−	?	+
Two distinct anal gland openings in males, found anteriorly to the anus	−	+	?	+	−	+	+	+	?	+

[1]Feature found in *Heteroperipatus clarki*, which might not belong to this genus (Oliveira *et al.* 2012).

[2]Feature, which is also found in peripatopsid species. However, the structure of the spinous pads is clearly different between Peripatidae and Peripatopsidae, as they are not socle-shaped and the second pad is always larger than the first and the third pads in the species of Peripatopsidae (see Read 1988a).

[3]The presence of ovarian funnels in this taxon has been shown by Bouvier (1905), Kemp (1914) and Reid (1996).

[4]The lack of the anterior ligament might be a dissection artifact since it is present in most Peripatidae and Peripatopsidae taxa (Mayer & Tait 2010). Only in representatives of *Metaperipatus* and *Peripatopsis* the ovary is fused along its entire length with the pericardial septum (Mayer 2007; Mayer & Tait 2010).

[5]Note that this feature has also been reported from different species of Peripatopsidae (Purcell 1899; Kemp 1914).

References

Arnett RH (1947) *Epiperipatus braziliensis* (Bouvier) on Barro Colorado Island, Canal Zone. Entomological News 58: 59–60.

Arnett RH (1961) The Onychophora of Jamaica. Entomological News 72: 213–220.

Bouvier EL (1905) Monographie des Onychophores. Annales des Sciences Naturelles, Zoologie et Biologie Animale [9e Série] 2: 1–383.

Brues CT (1911) A new species of *Peripatus* from Grenada, with observations on other species of the genus. Bulletin of the Museum of Comparative Zoology 54: 303–318.

Brues CT (1913) Preliminary descriptions of two new forms of *Peripatus* from Haiti. Bulletin of the Museum of Comparative Zoology 54: 517–521.

Brues CT (1914) A new *Peripatus* from Columbia. Bulletin of the Museum of Comparative Zoology 58: 375–382.

Brues CT (1917) A new species of *Peripatus* from the mountains of northern Peru. Bulletin of the Museum of Comparative Zoology 61: 383–387.

Brues CT (1935) Varietal forms of *Peripatus* in Haiti. Psyche 42: 58–62.

Brues CT (1939) Additional records of Onychophora from the island of Haiti. Psyche 46: 36–37.

Camerano L (1897) Nuova specie di *Peripatus* raccolta dal Prof. L. Balzan in Bolivia. Annali del Museo Civico di Storia Naturale di Genova Ser. II, v. 18: 12–15.

Camerano L (1898) Nouva specie di *Peripatus* dell'Ecuador. Atti Accademia delle Scienze di Torino 33: 308–310.

Carvalho ALd (1942) Sobre *Peripatus heloisae* do Brasil Central. Boletim do Museu Nacional, Nova Série, Zoologia 2: 57–89.

Clark AH (1913) A revision of the American species of *Peripatus*. Proceedings of the Biological Society of Washington 26: 15–19.

Clark AH (1937) On some onychophores from the West Indies and Central America. Proceedings of the United States National Museum 85: 1–3.

Cockerell TDA (1893) Notes on *Peripatus jamaicensis* , Grabh. and Ckll.. Zoologischer Anzeiger 16: 341–343.

Cockerell TDA (1901) The Jamaican species of *Peripatus*. Nature 63: 325–326.

Cockerell TDA (1908) Monographie des Onychophores by E.L. Bouvier. Science 27: 619–621.

Cockerell TDA (1913) A *Peripatus* from Guatemala. Proceedings of the Biological Society of Washington 26: 87–88.

du Bois-Reymond Marcus E (1952) On South American Malacopoda. Boletins da Faculdade de Filosofia, Ciências e Letras, Universidade de São Paulo, Série Zoologia 17: 189–209.

Dunn ER (1943) Zoological results of the Azuero Peninsula Panama Expedition of 1940. Part I — A new species of *Peripatus*. Notulae Naturae 123: 1–5.

Evans R (1901a) *Eoperipatus butleri* (nov. sp.). Quarterly Journal of Microscopical Science 44: 539–545.

Evans R (1901b) On two new species of Onychophora from the Siamese Malay States. Quarterly Journal of Microscopical Science 44: 473–538.

Evans R (1903) On *Peripatus guianensis* (sp. nov.). Quarterly Journal of Microscopical Science 47: 145–160.

Froehlich CG (1962) A *Peripatus* from Barbados. Boletim da Faculdade de Filosofia Ciências e Letras Universidade de São Paulo, Série Zoologia 261: 325–334.

Froehlich CG (1968) On some Brazilian onychophores. Beiträge zur Neotropischen Fauna 5: 160–171.

Fuhrmann O (1913) Über einige neue neotropische *Peripatus*-Arten. Zoologischer Anzeiger 42: 241–248.

Fuhrmann O (1915) Über eine neue *Peripatus*-Art vom Oberlauf des Amazonas. Abhandlungen der Senckenbergischen Naturforschenden Gesellschaft 36: 275–283.

Grube E (1853) Über den Bau von *Peripatus edwardsii.* Müller's Archives of Anatomy and Physiology [1853]: 322–360.

Guilding L (1826) Mollusca Caribbaeana. Zoological Journal 2: 437–449.

Jerez-Jaimes JH, Bernal-Pérez MC (2009) Taxonomía de onicóforos de Santander, Colombia y termogravimetría, calorimetría de barrido diferencial y espectroscopía infrarroja de la secreción adhesiva (Onychophora: Peripatidae). Revista de Biología Tropical 57: 567–588.

Kemp S (1913) Preliminary note on a new genus of Onychophora from the N. E. frontier of India. Records of the Indian Museum 9: 241–242.

Marcus E, Marcus E (1955) A new *Peripatus* from Minas Gerais, Brazil. Anais da Academia Brasileira de Ciências 27: 189–193.

Mayer G (2007) *Metaperipatus inae* sp. nov. (Onychophora: Peripatopsidae) from Chile with a novel ovarian type and dermal insemination. Zootaxa 1440: 21–37.
Mayer G, Tait NN (2009) Position and development of oocytes in velvet worms shed light on the evolution of the ovary in Onychophora and Arthropoda. Zoological Journal of the Linnean Society 157: 17–33.
Morera-Brenes B, León MD (1986) Description of *Macroperipatus valerioi* n. sp. from Costa Rica, and comments on the genus *Macroperipatus* (Onychophora: Peripatidae). Revista de Biología Tropical 34: 277–282.
Morera-Brenes B, Monge-Nájera J (1990) *Epiperipatus hilkae*, n. sp. from Costa Rica (Onychophora: Peripatidae). Revista de Biología Tropical 38: 449–455.
Morera-Brenes B, Monge-Nájera J (2010) A new giant species of placented worm and the mechanism by which onychophorans weave their nets (Onychophora: Peripatidae). Revista de Biología Tropical 58: 1127–1142.
Oliveira IS, Lacorte GA, Fonseca CG, Wieloch AH, Mayer G (2011) Cryptic speciation in Brazilian *Epiperipatus* (Onychophora: Peripatidae) reveals an underestimated diversity among the peripatid velvet worms. PLoS ONE 6: 13.
Oliveira IS, Read VMSTJ, Mayer G (2012) A world checklist of Onychophora (velvet worms), with notes on nomenclature and status of names. ZooKeys 211: 1–70.
Oliveira IS, Wieloch AH (2005) *Macroperipatus machadoi* sp. n. (Onychophora: Peripatidae) da floresta atlântica de Minas Gerais, Brasil. Lundiana 6 (supplement): 61–66.
Oliveira IS, Wieloch AH, Mayer G (2010) Revised taxonomy and redescription of two species of the Peripatidae (Onychophora) from Brazil: a step towards consistent terminology of morphological characters. Zootaxa 2493: 16–34.
Peck SB (1975) A review of the New World Onychophora with the description of a new cavernicolous genus and species from Jamaica. Psyche 82: 341–358.
Pocock RI (1894) Contributions to our knowledge of the arthropod fauna of the West Indies — Part III. Diplopoda and Malacopoda, with a supplement on the Arachnida of the class Pedipalpi. Journal of the Linnean SocietyZoology 24: 473–544.
Pollard EC (1893) Notes on the *Peripatus* of Dominica. Quarterly Journal of Microscopical Science 35: 285–293.
Purcell WF (1899) On the South African species of Peripatidae in the collection of the South African Museum. Annals of the South African Museum 1: 331–351.
Read VMSJ (1988a) The application of scanning electron microscopy to the systematics of the neotropical Peripatidae (Onychophora). Zoological Journal of the Linnean Society 93: 187–223.
Read VMSJ (1988b) The Onychophora of Trinidad, Tobago and the Lesser Antilles. Zoological Journal of the Linnean Society 93: 225–257.
Reid AL (1996) Review of the Peripatopsidae (Onychophora) in Australia, with comments on peripatopsid relationships. Invertebrate Taxonomy 10: 663–936.
Ruhberg H (1985) Die Peripatopsidae (Onychophora). Systematik, Ökologie, Chorologie und phylogenetische Aspekte. In: Schaller F (Ed) Zoologica, Heft 137. E. Schweizerbart'sche Verlagsbuchhandlung, Stuttgart.
Sclater WL (1888) On the early stages of the development of South American species of *Peripatus*. Quarterly Journal of Microscopical Science 28: 343–363.
Scorza JV (1953) Contribución al estudio de los *Peripatus* Caribes (*Epiperipatus*) de Venezuela, con adición de una nueva especie. Revista de sanidad y asistencia social 18: 783–788.
Sedgwick A (1888) A monograph on the species and distribution of the genus *Peripatus* (Guilding). Quarterly Journal of Microscopical Science 28: 431–493.
von Kennel J (1883) Entwicklungsgeschichte von *Peripatus.* Zoologischer Anzeiger 6: 531–537.
Wheeler WM (1898) A new *Peripatus* from Mexico. Journal of Morphology 15: 1–8.
Zilch A (1954a) Ein neuer *Oroperipatus* aus Peru. Senckenbergiana Biologica 35: 151–154.
Zilch A (1954b) Onychophoren aus El Salvador. Senckenbergiana Biologica 35: 147–150.
